# Risk stratification based on DNA damage-repair-related signature reflects the microenvironmental feature, metabolic status and therapeutic response of breast cancer

**DOI:** 10.3389/fimmu.2023.1127982

**Published:** 2023-03-24

**Authors:** Chunzhen Li, Shu Yu, Jie Chen, Qianshan Hou, Siyi Wang, Cheng Qian, Shulei Yin

**Affiliations:** National Key Laboratory of Medical Immunology and Institute of Immunology, Naval Medical University, Shanghai, China

**Keywords:** DNA damage, DNA repair, prognostic signature, immune microenvironment, metabolic status, breast cancer

## Abstract

DNA damage-repair machinery participates in maintaining genomic integrity and affects tumorigenesis. Molecular signatures based on DNA damage-repair-related genes (DRGs) capable of comprehensively indicating the prognosis, tumor immunometabolic profile and therapeutic responsiveness of breast cancer (BRCA) patients are still lacking. Integrating public datasets and bioinformatics algorithms, we developed a robust prognostic signature based on 27 DRGs. Multiple patient cohorts identified significant differences in various types of survival between high- and low-risk patients stratified by the signature. The signature correlated well with clinicopathological factors and could serve as an independent prognostic indicator for BRCA patients. Furthermore, low-risk tumors were characterized by more infiltrated CD8^+^ T cells, follicular helper T cells, M1 macrophages, activated NK cells and resting dendritic cells, and fewer M0 and M2 macrophages. The favorable immune infiltration patterns of low-risk tumors were also accompanied by specific metabolic profiles, decreased DNA replication, and enhanced antitumor immunity. Low-risk patients may respond better to immunotherapy, and experience improved outcomes with conventional chemotherapy or targeted medicine. Real-world immunotherapy and chemotherapy cohorts verified the predictive results. Additionally, four small molecule compounds promising to target high-risk tumors were predicted. *In vitro* experiments confirmed the high expression of GNPNAT1 and MORF4L2 in BRCA tissues and their association with immune cells, and the knockdown of these two DRGs suppressed the proliferation of human BRCA cells. In summary, this DNA damage-repair-related signature performed well in predicting patient prognosis, immunometabolic profiles and therapeutic sensitivity, hopefully contributing to precision medicine and new target discovery of BRCA.

## 1 Introduction

As the most commonly diagnosed malignancy globally, breast cancer (BRCA) has become a major driver of cancer-related deaths in women (1). Advances in early detecting techniques and multidimensional therapeutic modalities such as surgery, neoadjuvant chemoradiotherapy, and hormonal therapy have largely decreased the mortality of BRCA, but a proportion of patients still suffer from poor outcomes attributable to factors including tumor metastasis, recurrence, and drug resistance (2). Immunotherapy, as represented by adoptive cell therapy (ACT), tumor vaccines, and immune checkpoint blockade (ICB) therapy, etc., has added new perspectives and alternatives for cancer treatment. Nowadays, ICB agents such as Pembrolizumab and Atezolizumab have shown relatively satisfactory efficacy in treating triple-negative breast cancer (TNBC), especially in patients who were responsive (3–5). However, the response rate of patients receiving immunotherapy remains suboptimal, restricting patients from further benefit from immunotherapy (5). Therefore, identifying new prognostic indicators, therapeutic response predictors, and even promising molecular targets is of great relevance to further improve the prognosis of BRCA patients.

Genomic instability is one of the pervasive hallmarks of cancer (6). DNA damage and repair (DDR) mechanisms serve an indispensable role in the maintenance of genome stability. Accumulating evidence has indicated that DDR mechanisms contribute to regulating the oncogenesis, progression, genetic susceptibility, and therapeutic sensitivity of neoplasms (6, 7). Defects in DDR-related genes and resulting genomic instability are relevant to the malignant progression of breast cancer, leading to worsening disease and poor prognosis of patients (8, 9). It has been demonstrated that germline mutations in BRCA1 or BRCA2 are major drivers of the genetic susceptibility of BRCA (10). Poly (ADP-ribose) polymerase (PARP) inhibitors, capable of stunting the base excision repair function of PARP and promoting the formation of DSB (DNA double-strand breaks), leading to enhanced cell apoptosis, have shown effectiveness in treating patients with BRCA1 or BRCA2 mutations (8, 11). In addition, TNBC with homologous recombination repair (HRR) deficiency and BRCA1 methylation accompanied by low mRNA expression proved more sensitive to platinum-based agents (12). Therefore, the DDR pathway is a promising direction to explore prognostic biomarkers and interference targets in BRCA.

Accumulating evidence has demonstrated that tumor microenvironment (TME) constituents including infiltrative immune cells and stromal cells and even their secreted substances significantly influence therapeutic resistance and survival outcomes in BRCA patients (13–15). Microenvironmental status is associated with cancer progression along with alterations in the DDR pathway (16). In addition, the DDR pathway alterations of tumor cells can also affect the immune infiltration pattern and antitumor immune response by modulating the immune checkpoint expression and immune recognition of tumor cells (17, 18). Importantly, combinational therapy with DDR inhibitors and ICB agents significantly increased infiltrative lymphocytes and augmented T cell-mediated immune killing and antitumor immunity against cancer cells (18–21). Hence, the investigation of the interaction between the tumor DDR signaling and TME could facilitate adequate uncovering of the value of DDR-related molecules as prognostic and therapeutic biomarkers for BRCA patients.

This study presents a DNA damage-repair-related prognostic signature performing well in indicating patient prognosis, microenvironmental features, and therapeutic preferences. BRCA patients stratified by this signature exhibited remarkably different clinicopathological outcomes, tumor immune microenvironment landscapes, antitumor immune function, and therapeutic sensitivity, hopefully yielding novel insights into the target discovery and precision medicine of BRCA.

## 2 Materials and methods

### 2.1 Data collection

From the Cancer Genome Atlas (TCGA) database (https://portal.gdc.cancer.gov/) we obtained RNA-seq data and clinical information of 1109 BRCA samples and 113 normal samples. The Molecular Signatures Database (MSigDB, https://www.gsea-msigdb.org/gsea/msigdb/) and published studies were used to collect DNA damage-repair-related genes (DRGs) (22–24). For signature validation, two independent datasets (GSE20685 and GSE96058) were downloaded from the Gene Expression Omnibus (GEO) database (https://www.ncbi.nlm.nih.gov/geo). GSE20685 dataset includes RNA expression and clinical data such as the recurrence and metastasis information of 327 BRCA patients. The RNA-seq, tumor size, histological grade, lymph node involvement, as well as survival data of 3273 BRCA patients in the GSE96058 dataset were also collected from the GEO database. More clinical information about the three cohort was shown in **Supplementary Table S1**.

### 2.2 Screening of prognostic DRGs and visualizing the interaction network

Firstly, we extracted differentially expressed DRGs through running the R package “limma”, with parameters set as log_2_|(fold change (FC))| > 0.585 and FDR < 0.001. Subsequently, Gene Ontology (GO) and Kyoto Encyclopedia of Genes and Genomes (KEGG) enrichment analyses were conducted. RNA expression data were merged with survival information of patients, while duplicates and samples with an overall survival time of less than 60 days were removed, so that 1023 samples were used in the prognostic analysis. Prognostic DRGs were screened from differentially expressed DRGs utilizing univariate Cox regression. The STRING database (https://cn.string-db.org/) provided the interactions of prognostic DRGs, those with scores less than 0.4 and disconnected nodes are removed (25). Differentially expressed transcriptome factors (TFs) associated with prognostic DRGs (determined as |correlation| > 0.3 & *P* < 0.001) were also screened and the Cytoscape software was applied to visualize the TF-gene network (26).

### 2.3 Establishment and validation of the signature

TCGA-BRCA cohort (N = 1023) worked as the training cohort, and datasets GSE96058 (N = 3273) and GSE20685 (N = 327) were used as testing cohorts for external validation. LASSO (Least Absolute Shrinkage and Selection Operator) process was carried out in TCGA-BRCA cohort for further determination of the DRGs used for signature establishment and their coefficients. Formula for calculating the risk score was: $Risk\;score=\sum_{i=1}^{n}Coefficient\left(i\right)*Expression\left(i\right)$ . Then every patient was given a risk score. In accordance with the median of calculated scores, the TCGA-BRCA cohort was divided into high-risk and low-risk groups. It also served as the grouping cutoff for validation cohorts. Using the R packages “survival” and “survminer”, we plotted the Kaplan-Meier curves for the comparison of survival outcomes. The “pheatmap” package was applied to display the expression of signature DRGs. To assess the prognostic accuracy of the signature, the time-dependent receiver operating characteristic (ROC) curves were drawn.

### 2.4 Clinicopathological significance of the signature and development of the predictive nomogram

Patients in the training cohort were further divided into subgroups according to the PAM50 subtypes of BRCA, and survival analyses were performed to confirm the stability of this signature in patients with different PAM50 subtypes. Comparisons of the outcomes between patients with different risk were also carried out, and different types of survival such as disease-free survival (DFS), progression-free survival (PFS), disease-specific survival (DSS), metastasis-free survival (MFS) and recurrence-free survival (RFS) were observed. Then the univariate and multivariate regressions were conducted for assessing whether this signature along with other clinicopathological variables were capable of indicating the prognosis of BRCA patients independently. Independent prognostic indicators were further extracted for developing the predictive nomogram. The predictive accuracy of the nomogram was evaluated using ROC and calibration curves.

### 2.5 Profile of the metabolism, DNA repair and cancer-related characteristics

To investigate the metabolic status of tumors in different risk groups, we calculated various metabolic process scores for each sample using the “IOBR” package, a functional package for decoding the oncological and biological features of tumors based on GSVA (Gene Set Variation Analysis) and multiple integrated signatures (27). We also incorporated cancer-related features such as DNA mismatch repair, hypoxia, apoptosis, ferroptosis, exosome, epithelial-mesenchymal transition (EMT) and angiogenesis to fully investigate the differences in functional phenotypes (27–29).

### 2.6 Gene set enrichment analysis

Gene set enrichment analysis (GSEA) was carried out to search for significantly enriched pathways in risk groups stratified by the signature to suggest putative molecular mechanisms. The “gmt” files for the KEGG and GO gene sets were downloaded from the MSigDB. R packages “org.Hs.eg.db” and “clusterProfiler” were adopted to conduct the analysis. Five significantly enriched terms or pathways in each group were selected and visualized.

### 2.7 Dissection of the tumor microenvironment

Multiple algorithms were applied to characterize the TME in this section. Proportions of tumor-infiltrating immune cells along with their correlation with the signature score were analyzed using the xCELL, CIBERSORT and MCPCOUNTER platforms. Differences in the abundance of 22 immune cell subtypes calculated by CIBERSORT between risk groups were shown. Moreover, the relationship between the expression of DRGs constituting the signature and the CIBERSORT immune cells was analyzed based on the Spearman correlation method. With the R package “estimate”, we calculated microenvironmental scores including stromal score and immune score, etc. We also compared RNA expression-based stemness scores (RNAss) between risk groups. Thorsson et al. reported immunogenomic landscapes of multiple cancers in TCGA database, and we introduced data on the intra-tumor heterogeneity and proliferation scores of BRCA patients derived from their team (30). To gain a deeper understanding of the differences in factors associated with antitumor immunity, we conducted the GSVA analysis based on “IOBR” packages after selecting several gene sets reported previously (27, 31, 32).

### 2.8 Identifying patient sensitivity to immunotherapy and chemotherapy

Firstly, we analyzed the expression of antitumor immunity-related molecules including major histocompatibility complex (MHC), immune coinhibitory molecules and immune costimulatory molecules. TIDE (Tumor Immune Dysfunction and Exclusion) and IPS (Immunophenoscore) algorithms were applied to quantify characteristics such as tumor immunogenicity and immune evasion to further evaluate the effectiveness of immunotherapy for specific patients (33, 34). Multiple scores such as the TIDE score and interferon score calculated by the TIDE algorithm, as well as the IPS score, together with several antitumor immunity-related signatures from previous publications, provided explanations for the differences in responsiveness to immunotherapy between patients. The sensitivity of each sample to common chemical agents clinically used, including Cisplatin, Paclitaxel and Doxorubicin et al., was represented by the IC50 (the half-maximal inhibitory concentration) values calculated by the “pRRophetic” package (35). In order to further validate the predictive results, we included transcriptomic and clinical data of real-world patient cohorts receiving chemotherapy (GSE4779, GSE20271 and GSE59515) or immunotherapy (GSE78220, GSE91061, GSE126044 and GSE35640).

The Connectivity Map (Cmap, https://clue.io/) platform was further applied to screen drugs that might target high-risk tumors. After completing the differential analysis between risk groups, genes with |log_2_FC| > 1 and FDR < 0.05 were retrieved. And expression alterations of those genes were uploaded to the Cmap platform to predict effective compounds for high-risk tumors (36). Utilizing the Pubchem website (http://pubchem.ncbi.nlm.nih.gov/), structures of those effective agents were displayed (37).

### 2.9 Proteomic expression of DRGs associated with clinicopathological features

After comparing the differences in mRNA levels of these DRGs under different clinicopathological features, we utilized multi-omics data from publicly available databases together with experimental approaches to validate the expression of DRGs associated with clinicopathological factors in tumoral and noncancerous tissues to see whether the differences in protein levels were consistent with those mRNA results above. The Human Protein Atlas (HPA) database (https://www.proteinatlas.org/) provides immunohistochemistry (IHC) data, and the Clinical Proteomic Tumor Analysis Consortium (CTPAC) database (https://pdc.cancer.gov/pdc/) was available for proteomic data (38, 39). We also carried out quantitative real-time PCR (qRT-PCR) and IHC analyses using clinical BRCA samples.

### 2.10 Cell culture and tissue samples

Human breast cancer cell line MCF-7 were obtained from the American Type Culture Collection. Cells were cultured in DMEM (Gibco) supplemented with 10% FBS (Gibco) at 37°C with 5% CO_2_. Five pairs of freshly resected BRCA tissues and corresponding adjacent normal tissues were obtained from the Department of Thyroid and Breast Surgery, Shanghai Changhai Hospital, with the informed consents of the patients. Fresh tissues were snap frozen and stored in liquid nitrogen until RNA extraction.

### 2.11 RNA extraction and quantitative real-time PCR

Total RNA was extracted from cultured cells or tissue samples using TRIzol reagent in accordance with the manufacturer’s instructions. Reverse transcription of RNA to cDNA was performed using TaKaRa’s reverse transcription reagents (Oligo (dT) primer and M-MLV Reverse Transcriptase) following standard procedures. RNA expression was quantified by real-time PCR with TB Green Premix Ex Taq (TaKaRa) and normalized by the level of GAPDH. Relative expression level is calculated using the 2^−ΔΔCt^ method. All primers used were synthesized by Sangon Biotech (Shanghai, China). The sequences of the primers were listed in **Supplementary Table S2**.

### 2.12 Cell transfection and cell counting Kit-8 assay

Small interfering RNAs (siRNAs) targeting the human GNPNAT1 and MORF4L2 were obtained from Genepharma (Shanghai, China). The sequences were listed in **Supplementary Table S2**. MCF-7 cells were transfected with siRNAs (20nM) at confluency of 30–50% using the Lipofectamine RNAiMAX Reagent (Invitrogen). The Opti-MEM (Invitrogen) was utilized as the medium diluting siRNA and transfection reagents, and subsequent experiments such as RNA extraction and quantitative PCR and cell proliferation viability assay were performed 48 hours after transfection. We investigated the effect of GNPNAT1 and MORF4L2 knockdown on the proliferation ability of BRCA cells by cell counting kit-8 (CCK-8) assay. Each group of cells was inoculated in a 96-well plate (2×10^3^ cells per well). The CCK-8 reagent (Vazyme) was added at 0h, 24h, 48h and 72h after cell adhesion, and the absorbance measurements were performed according to the manufacturer’s instructions.

### 2.13 Immunohistochemistry staining

The protein abundance of GNPNAT1 and MORF4L2 in BRCA and normal paracancerous tissues was detected using IHC. Primary antibodies against GNPNAT and MORF4L2 were purchased from Proteintech and Origene, respectively. Antibodies against CD8 alpha and CD68 were provided by Servicebio. The tissue samples were fixed with 4% paraformaldehyde and then dehydrated, fixed, embedded and sliced following standard procedures. Slides were incubated with primary antibodies at 4°C overnight. Before incubating the secondary antibody, wash the slides three times with PBS. Add the secondary antibody and incubate for 1 hour at room temperature, protected from light. The nuclei were stained using DAPI. Finally, microscopic imaging and image acquisition were performed.

### 2.14 Software and statistical methods

Most of the bioinformatic analyses mentioned in this research were achieved *via* R software (version 4.1.2), except for some online website-based analyses such as the compound prediction. A variety of R packages including “limma”, “ggpubr”, “pheatmap”, “GSVA”, “survival”, “IOBR”, “estimate” and “pRRophetic”, etc. were implemented in this study. The Student’s t-test was used to evaluate continuous variables, whereas the χ2 test was used to compare categorical variables. The Wilcoxon test was used to compare the differences in gene expression between groups. Analysis of correlation between variables was performed using Spearman and Pearson methods. GraphPad Prism (version 9.0.0) was adopted to generate the experimental analyses. For all statistical analyses, a p-value less than 0.05 was regarded as statistical significance.

## 3 Results

### 3.1 Identification of DNA damage and repair-related genes with prognostic value

**Figure 1:**
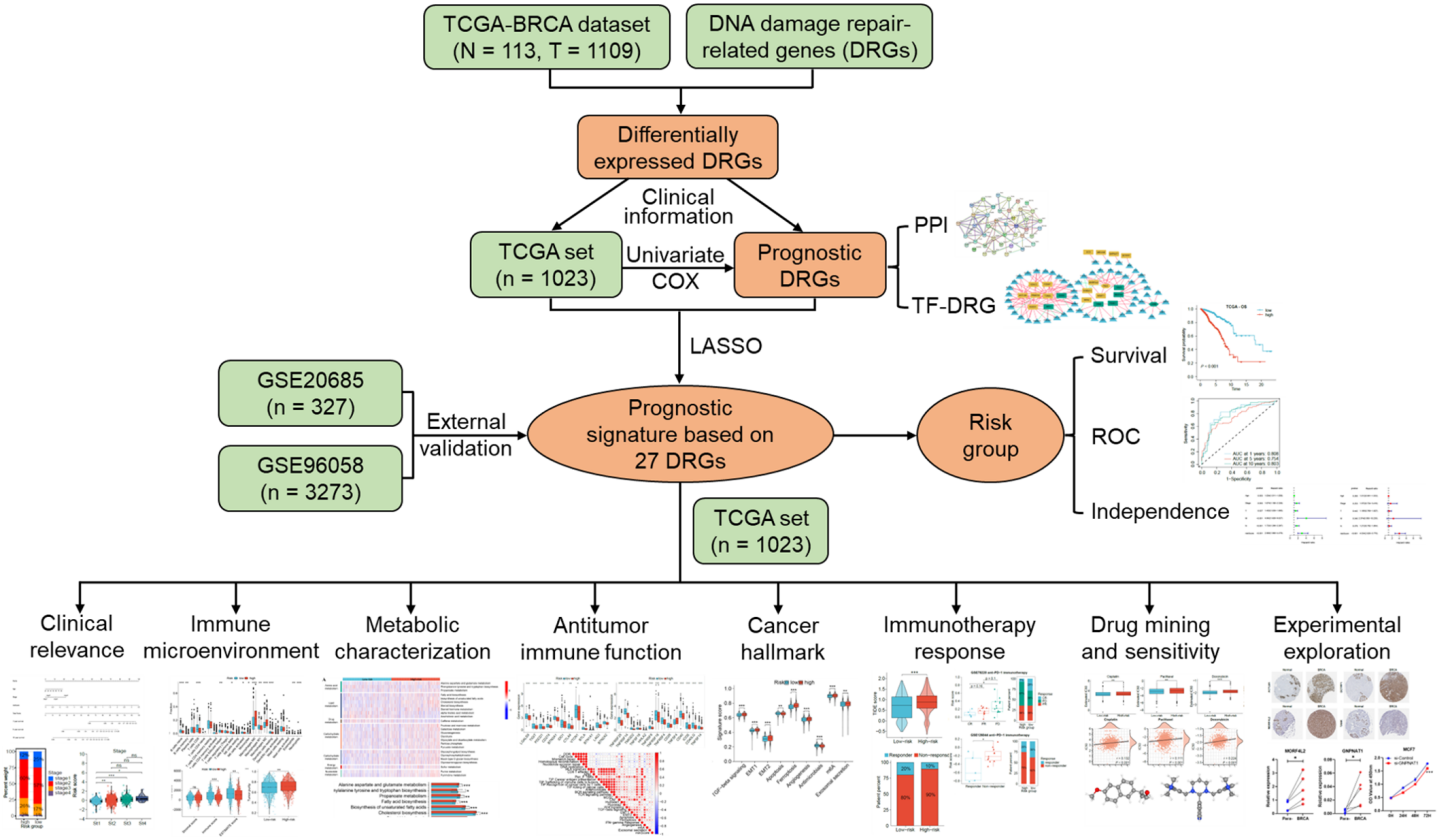
The overall framework of this research.

**Figure 2:**
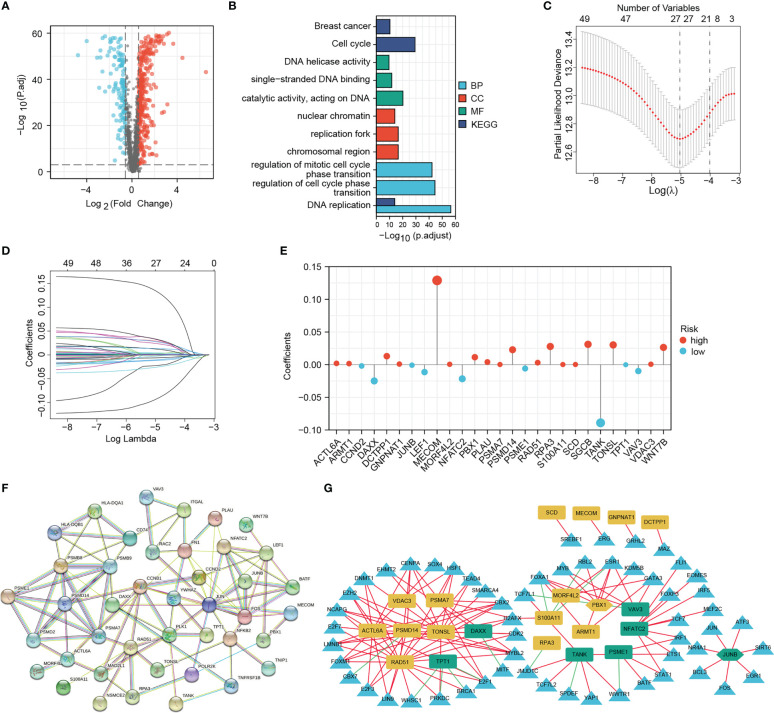
Screening for differentially expressed DNA damage and repair-related genes (DRGs) for prognostic signature establishment. **(A)** Volcano map of the differentially expressed DRGs. **(B)** GO and KEGG enrichment results of differentially expressed DRGs. **(C–E)** LASSO analysis confirmed the candidate DRGs and their coefficients. **(F)** PPI network of prognostic DRGs. **(G)** Interaction network of signature DRGs and their related TFs; The red and green lines represent positive and negative correlations, respectively. The yellow and green graphs represent high-risk and low-risk MRGs, respectively. Triangles represent TFs. Two hexagons represent TFs that also act as signature DRGs.

The overall framework of this study is illustrated in **Figure 1**. The collected DRGs were intersected and de-duplicated with the genes in TCGA-BRCA expression data, totaling 1581 DRGs were obtained for subsequent analyses. According to the criteria of |log_2_(FC)| > 0.585 and *P* < 0.001, a total of 448 differentially expressed DRGs were identified, of which 128 were down-regulated and 320 were up-regulated in BRCA tissues (**Figure 2A**). They are mainly enriched in biological pathways associated with breast cancer, cell cycle and DNA replication, molecular functions such as DNA strand uncoiling and binding, and cellular components including chromatin and replication forks (**Figure 2B**). After the univariate Cox regression, fifty-one prognostic DRGs were obtained (*P* < 0.05), among which 27 were hazardous (Hazard ratio (HR) > 1) and 24 were benign (HR < 1) (**Supplementary Table S3**).

### 3.2 Establishment of the DNA damage-repair-related prognostic signature

Based on 51 prognostic DRGs, we conducted LASSO analysis to determine the DRGs and their coefficients used for signature establishment (**Figures 2C, D**). Subsequently 27 DRGs including 9 low-risk genes and 18 high-risk genes merged from the list and their coefficients were shown in **Figure 2E** and **Table 1**. Additionally, for more clarity on the interactions of these molecules, the protein-protein interaction (PPI) and TF-gene regulatory network were introduced. We drew a PPI network using those 51 prognostic DRGs (**Figure 2F**). After correlation analysis of differential TFs with 27 signature DRGs, a network containing 22 signature DRGs (including two TFs, PBX1 and JUNB) and 57 DRG-related TFs was constructed (**Figure 2G**). Among them, the interactions between JUNB and JUN, as well as PBX1 and MYB have been investigated, so this regulatory network may bring ideas for understanding the regulation of DRG expression (40, 41).

**Table 1:** Coefficients of the signature DRGs.

Gene Symnol	Description	Coefficient
MECOM	MDS1 And EVI1 Complex Locus	0.12893
RPA3	Replication Protein A3	0.02782
MORF4L2	Mortality Factor 4 Like 2	0.00056
ACTL6A	Actin Like 6A	0.00199
NFATC2	Nuclear Factor of Activated T Cells 2	-0.02164
S100A11	S100 Calcium Binding Protein A11	0.00021
VDAC3	Voltage Dependent Anion Channel 3	0.00066
PSMD14	Proteasome 26S Subunit, Non-ATPase 14	0.02283
TANK	TRAF Family Member Associated NFKB Activator	-0.08903
WNT7B	Wnt Family Member 7B	0.02638
TONSL	Tonsoku Like, DNA Repair Protein	0.03017
VAV3	Vav Guanine Nucleotide Exchange Factor 3	-0.00981
PLAU	Plasminogen Activator, Urokinase	0.00402
GNPNAT1	Glucosamine-Phosphate N-Acetyltransferase 1	0.00092
PSME1	Proteasome Activator Subunit 1	-0.00594
JUNB	JunB Proto-Oncogene, AP-1 Transcription Factor Subunit	-0.00079
LEF1	Lymphoid Enhancer Binding Factor 1	-0.01138
RAD51	RAD51 Recombinase	0.00310
PSMA7	Proteasome 20S Subunit Alpha 7	0.00026
DAXX	Death Domain Associated Protein	-0.02490
PBX1	PBX Homeobox 1	0.01142
DCTPP1	DCTP Pyrophosphatase 1	0.01298
CCND2	Cyclin D2	-0.00200
SCD	Stearoyl-CoA Desaturase	0.00025
ARMT1	Acidic Residue Methyltransferase 1	0.00163
TPT1	Tumor Protein, Translationally-Controlled 1	-0.00012
SGCB	Sarcoglycan Beta	0.03111

**Figure 3:**
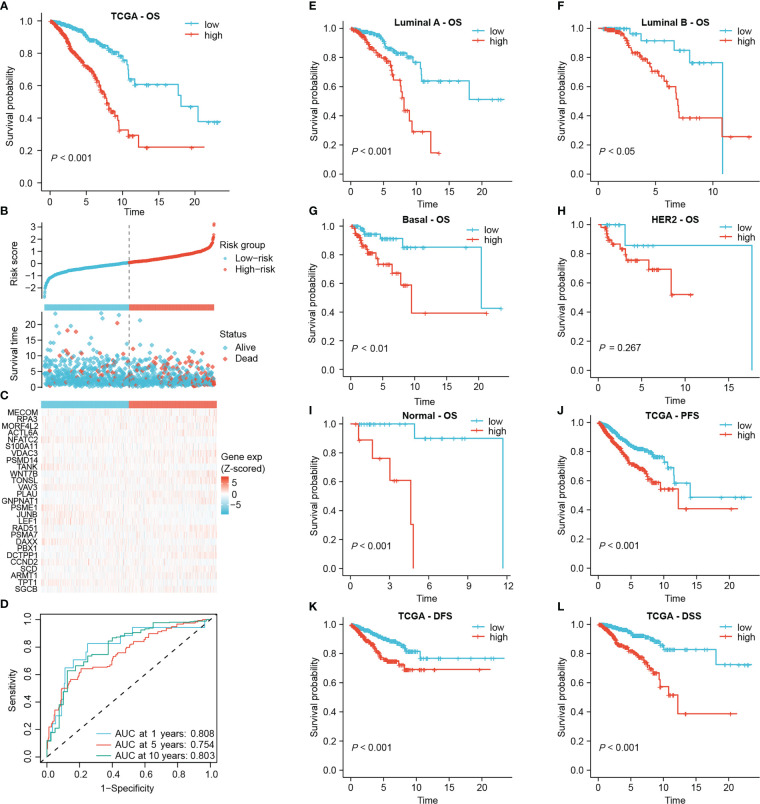
Establishing the prognostic signature using the TCGA-BRCA cohort. **(A)** Kaplan-Meier overall survival curve of all patients. **(B)** Risk score and survival status of each patient. **(C)** Heat map of signature DRG expression. The color from blue to red represents the gene expression from low to high. **(D)** ROC curves for signature evaluation. **(E–I)** Kaplan-Meier overall survival curves of patients with different PAM50 types. PFS curve **(J)**, DFS curve **(K)** and DSS curve **(L)** of all patients.

Subsequently, the risk score was calculated for each sample based on the coefficients and expression of the 27 DRGs involved in the signature. The median risk score in the TCGA-BRCA cohort was 0.83425, which classified 511 low-risk and 512 high-risk patients (**Figure 3B**). There was an obvious overall survival (OS) benefit for low-risk patients as compared to those high-risk individuals (*P* < 0.001, **Figure 3A**). In **Figure 3B, C**, patients’ risk scores and survival status were shown along with the 27 DRG expression among different risk groups. The area under the curve (AUC) of the 1-year, 5-year and 10-year ROC curves was 0.808, 0.754 and 0.803, respectively (**Figure 3D**). The signature was still capable of distinguishing OS differences between high- and low-risk individuals with Luminal A, Luminal B, Basal and Normal subtypes (*P* < 0.05) (**Figures 3E–I**). Yet, unfortunately, among patients with HER2 subtype, no statistically significant differences in OS were observed (*P* = 0.267, **Figure 3H**). In addition, after subgrouping patients based on their clinicopathological characteristics, high-risk patients still had a poorer overall prognosis, suggesting that the prognostic role of the signature was not vulnerable to clinicopathological factors (**Supplementary Figure S1A**). Moreover, high-risk patients experienced worse progression-free survival (PFS), disease-free survival (DFS), and disease-specific survival (DSS) than those of low-risk patients (**Figures 3J–L**).

### 3.3 Clinicopathological relevance of the signature and development of the predictive nomogram

**Figure 4:**
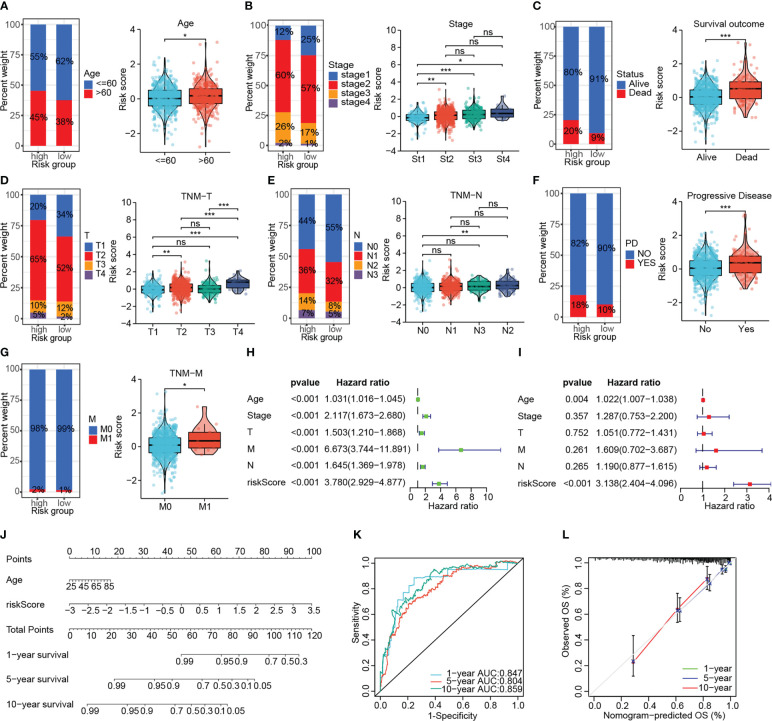
Investigating the association of the signature with the clinicopathological characteristics of patients in the TCGA-BRCA cohort. The association of the signature with the age **(A)**, AJCC stage **(B)**, survival outcome **(C)**, AJCC T-stage **(D)**, AJCC N-stage **(E)**, disease status **(F)** and AJCC M-stage **(G)**. **(H, I)** Univariate and multivariate Cox analyses confirmed the independent prognostic role of the signature. **(J)** The predictive nomogram containing the risk score and another independent prognostic factor. **(K, L)** The ROC curve and calibration curve to assess the predictive accuracy of the nomogram. (ns: not significant, *P < 0.05, **P < 0.01, ***P < 0.001).

Risk stratification based on DNA damage-repair-related genes correlated well with the clinicopathological characteristics of patients. The elderly patients had higher risk scores compared to those younger ones (*P* < 0.05, **Figure 4A**). We in turn investigated the correlation of the signature with AJCC stage, survival outcome, TNM stage, and disease progression status in BRCA patients, which revealed that high-risk patients had more advanced tumors, worse survival as well as treatment outcomes (**Figures 4B–G**). Cox regression determined the independence of age and risk score as prognosis predictors for overall survival from multiple clinical indicators, and the risk score showed the highest HR **(** **Figures 4H, I**). Subsequently, the nomogram incorporating two independent prognostic indicators for quantitative prediction of OS were constructed (**Figure 4J**). Time-dependent ROC and calibration curves show higher efficiency of the signature-derived nomogram in predicting patient OS (**Figures 4K, L**). Furthermore, the independent prognostic role of the signature on PFS, DFS and DSS was also demonstrated (**Supplementary Figure S2**).

### 3.4 The prognostic value of the signature was validated in two independent cohorts

**Figure 5:**
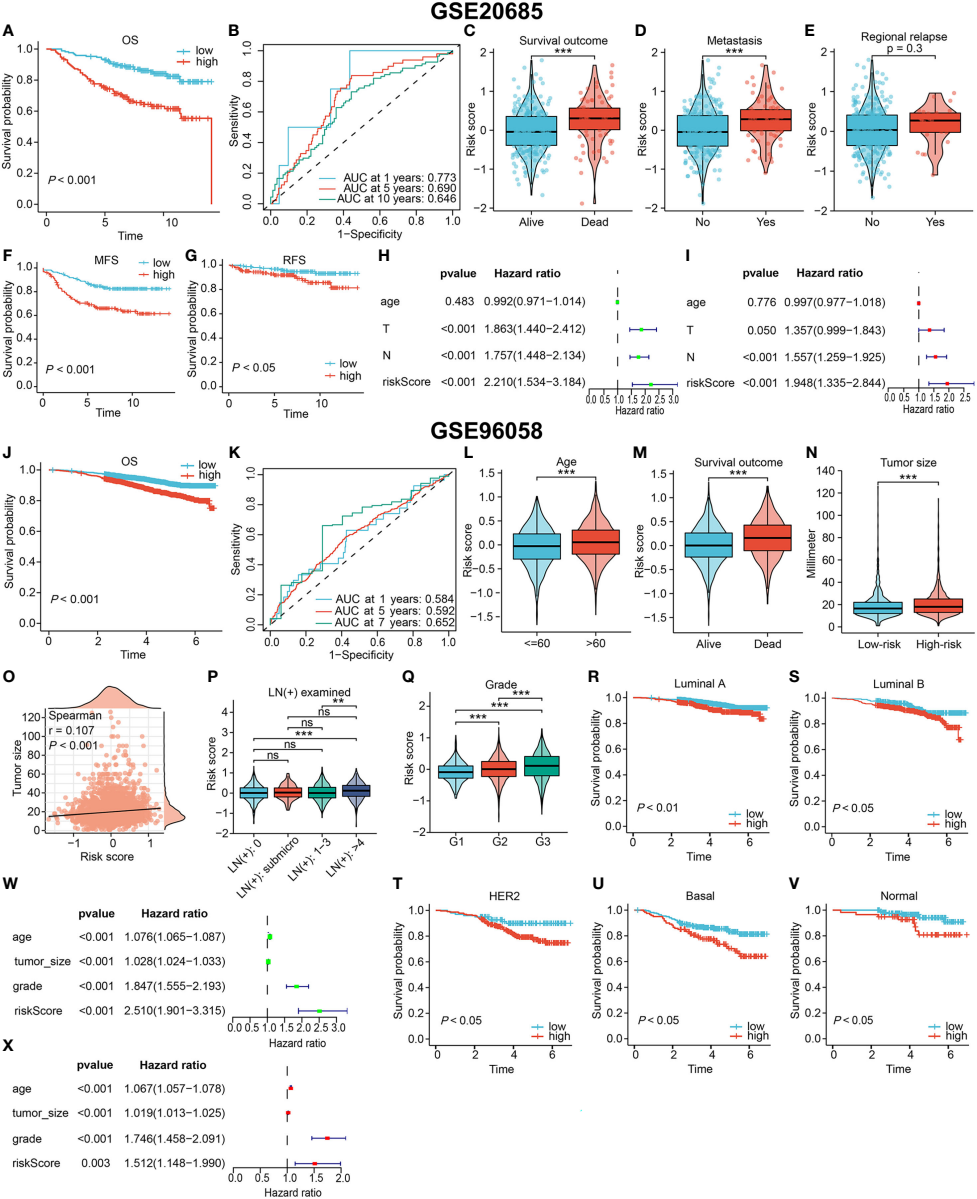
Validating the prognostic value in two independent external cohorts. **(A–I)** Results based on GSE20685 cohort. The OS curve **(A)**, MFS curve **(F)**, RFS curve **(G)** of patients. **(B)** The ROC curve to evaluate the predictive accuracy. The association of the signature with the survival outcome **(C)**, metastasis event **(D)** and relapse event **(E)**. Confirming the signature independence *via* univariate **(H)** and multivariate **(I)** Cox analyses. **(J–X)** Results based on GSE20685 cohort. **(J)** The OS curve of all patients. **(K)** The ROC curve to evaluate the predictive accuracy. The association of the signature with the age **(L)**, survival outcome **(M)**, tumor size **(N, O)**, lymph node involvement **(P)** and histological grade **(Q)**. **(R–V)** Overall survival curves of patients with different PAM50 types. Confirming the independent prognostic role *via* univariate **(W)** and multivariate **(X)** Cox analyses. (ns, not significant, **P < 0.01, ***P < 0.001).

The value of the DRG-based signature in indicating prognosis was verified in two external cohorts (GSE20685 and GSE96058), as evidenced by a shorter OS time for high-risk patients (*P* < 0.001, **Figures 5A, J**). And the signature maintained a satisfactory prognostic performance across different clinicopathological subgroups in two external cohorts (**Supplementary Figures S1B, C**). In the GSE20685 cohort, The AUC of the 1-year, 5-year and 10-year ROC curves were 0.773, 0.690 and 0.646, respectively (**Figure 5B**). Since few patients in the GSE96058 cohort were followed for more than 7 years, we specified the time-dependent ROC curve to year 7. The signature also showed good prediction in the GSE96058 cohort (**Figure 5K**). Patients from the GSE20685 cohort who experienced death or tumor metastasis events had higher risk scores (*P* < 0.001, **Figures 5C, D**). Risk scores were also higher but not statistically significant in patients with tumor relapse (*P* = 0.3, **Figure 5E**). Moreover, the signature was as effective in differentiating metastasis-free survival (MFS) and relapse -free survival (RFS) in high- and low-risk patients (**Figures 5F, G**). In the GSE96058 cohort, patients with age > 60, more detected positive lymph nodes (LNs), or death outcomes had higher risk scores (**Figures 5L, M, P**). High-risk patients were found to have larger tumor, and there was a correlation between the risk score and tumor size (**Figures 5N, O**). The positive correlation between the risk score and the histological grade of the tumor was significant (*P* < 0.001, **Figure 5Q**). In addition, the OS of high-risk patients was consistently worse than that of low-risk patients, regardless of the PAM50 subtype (all *P* < 0.05, **Figures 5R–V**). The independent prognostic performance of the signature was also verified in these two cohorts (**Figures 5H, I W, X**, respectively).

### 3.5 Identification and protein expression of DRGs associated with pathological progression

**Figure 6:**
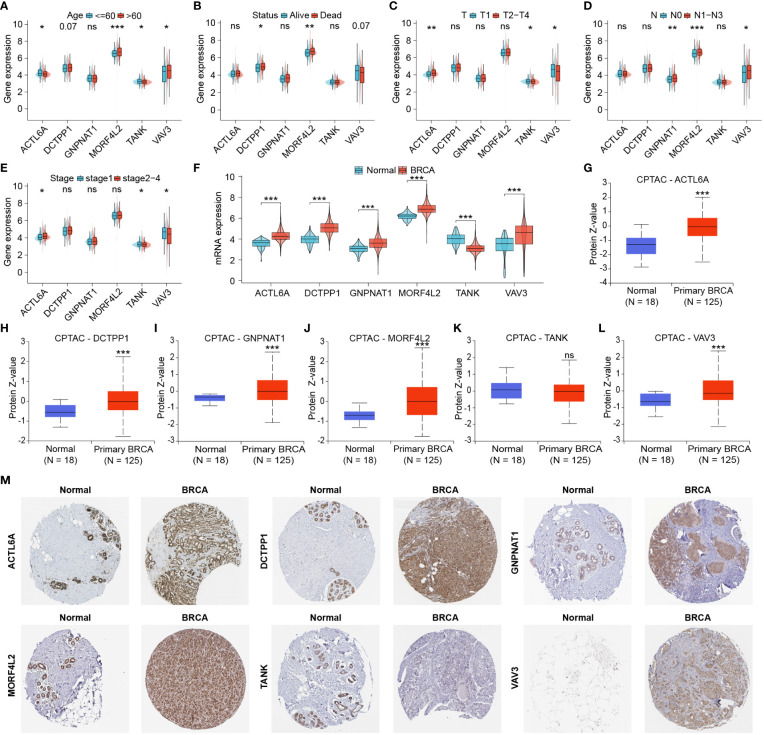
Identification and protein expression of DRGs associated with clinicopathological features. **(A–E)** DRGs differentially expressed in different clinicopathological subgroups. **(F)** Differential mRNA expression of six DRGs associated with clinicopathological features in normal and tumor tissues. **(G–L)** Proteomic expression of six DRGs based on CPTAC database. **(M)** Immunohistochemical images derived from the HPA database showed the levels of six proteins in normal and tumor tissues. (ns: not significant, *P < 0.05, **P < 0.01, ***P < 0.001).

For further exploration of DRGs associated with clinicopathological characteristics of BRCA patients, we analyzed differential expression of DRGs involved in this signature between different clinicopathological subgroups. Higher expression of MORF4L2 was found in patients with age > 60, poor survival outcomes, and higher lymph node (N) stage (**Figures 6A, B, D**). ACTL6A expression was higher in patients with advanced T stage and AJCC stage and lower in older patients. Both TANK and VAV3 were differentially expressed between subgroups based on age, T-stage, and AJCC stage (**Figures 6A, C, E**). DCTPP1 and GNPNAT1 were highly expressed in patients with death outcome and patients with higher N stage, respectively (**Figures 6B, D**). We then checked the differential mRNA expression of those 6 DRGs in noncancerous and tumor tissues, and found that the other five DRGs with high expression in BRCA tissues except for TANK with low expression (**Figure 6F**). Besides, we explored the proteomic level differences of these six DRGs in normal and BRCA tissues *via* CPTAC database and HPA database. **Figures 6G–L** revealed that the levels of five proteins, ACTL6A, DCTPP1, GNPNAT1, MORF4L2 and VAV3 were significantly upregulated in BRCA tissues, and the level of TANK protein was downregulated, but the difference was not statistically significant. Immunohistochemical images derived from the HPA database suggested that the levels of these six proteins in normal and BRCA tissues were basically in keeping with those RNA results from TCGA and proteomic results from CPTAC (**Figure 6M**).

### 3.6 Landscape of tumor-infiltrating immune cells

**Figure 7:**
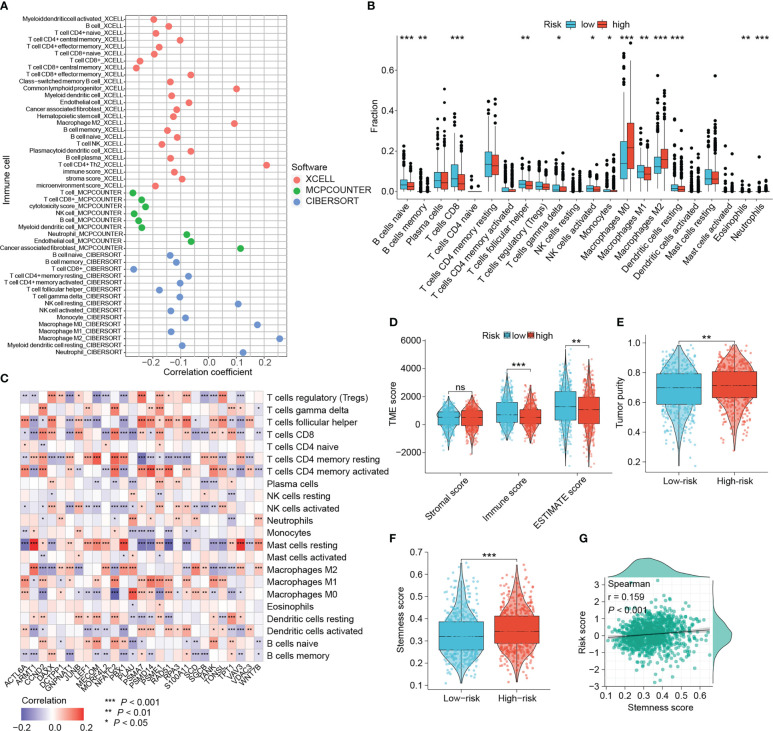
Dissection of the tumor microenvironment. **(A)** The correlation between the signature and tumor-infiltrating immune cells estimated by three algorithms. **(B)** Differential immune cells based on CIBERSORT algorithm between risk groups. **(C)** The relevance between the DRG expression and microenvironmental immune cells based on CIBERSORT algorithm. **(D, E)** Differences in microenvironmental scores and tumor purity. **(F, G)** Relationship between the signature score and tumor stemness. (ns: not significant, *P < 0.05, **P < 0.01, ***P < 0.001).

With growing evidence that DNA damage and repair could affect tumor immunogenicity, immune infiltration patterns, and be involved in modulating intrinsic and adaptive immune responses, we wanted to investigate the relevance between this DRG-based risk signature and tumor-infiltrating immune cells (42, 43). The immune infiltration patterns were characterized using three different algorithms (xCELL, MCPcounter and CIBERSORT). **Figure 7A** showed that the risk scores were negatively correlated with the amount of CD8^+^ T cells, the main subset of T cells that exert antitumor immunity. While the abundance of tumor-associated macrophages (TAMs), particularly M2 macrophages that suggest poor prognosis of BRCA patients, correlated positively with the risk score (**Figure 7A**). Consistently, higher levels of M0 and M2 macrophages, and lower levels of CD8^+^ T cells were found in the tumors of high-risk patients (**Figure 7B**). Moreover, there were more naïve B cells, follicular helper T cells, activated NK cells, monocytes, M1 macrophages and resting dendritic cells in low-risk tumors (**Figure 7B**).

With the results of the CIBERSORT algorithm, we wanted to explore the association between signature DRGs and the abundance of infiltrative immune cells. The expression of six DRGs, CCND2, DAXX, JUNB, NFATC2, PSME1 and TPT1, presented a strong positive correlation with CD8^+^ T cells (**Figure 7C**). In contrast, eight DRGs (ARMT1, GNPNAT1, MORF4L2, PBX1, PLAU, SCD, SGCB and VAV3) exhibited a strong negative association with CD8^+^ T cells (**Figure 7C**). The positive correlations of five DRGs (ARMT1, MORF4L2, PBX1, VAV3 and WNT7B) with M2 macrophages and resting mast cells were also observed. Six progression-related DRGs showed distinct correlation with immune cells. ACTL6A was positively correlated with follicular helper T cells, activated CD4^+^ memory T cells, M1 macrophages and M0 macrophages, while it was negatively correlated with resting mast cells. The three progression-related DRGs, GNPNAT1, MORF4L2 and VAV3, consistently correlated negatively with CD8^+^ T cells and positively with M2 macrophages. TANK expression was positively associated with multiple T cell subtypes (CD8^+^ T cells, activated CD4^+^ T cells, etc.) except for Treg. Interestingly, five DRGs (CCND2, NFATC2, S100A11, TANK and TONSL) showed inverse correlations with different macrophage phenotypes, indicating that they may influence macrophage polarization. These results show that both the signature and the DRGs used for developing the signature exhibit good relevance to immune cells.

There was no difference in stromal scores between the two groups, while immune scores and ESTIMATE scores were elevated in low-risk patients (*P* < 0.01, **Figure 7D**). Conversely, high-risk tumors were purer and also characterized by stronger cancer-stem-cell (CSC) properties (all *P* < 0.01, **Figures 7E, F**). Moreover, risk scores showed a significant positive correlation with cancer-stem-cell scores (*P* < 0.001, **Figure 7G**).

### 3.7 Distinct metabolic profiles and cancer hallmarks between the risk groups

**Figure 8:**
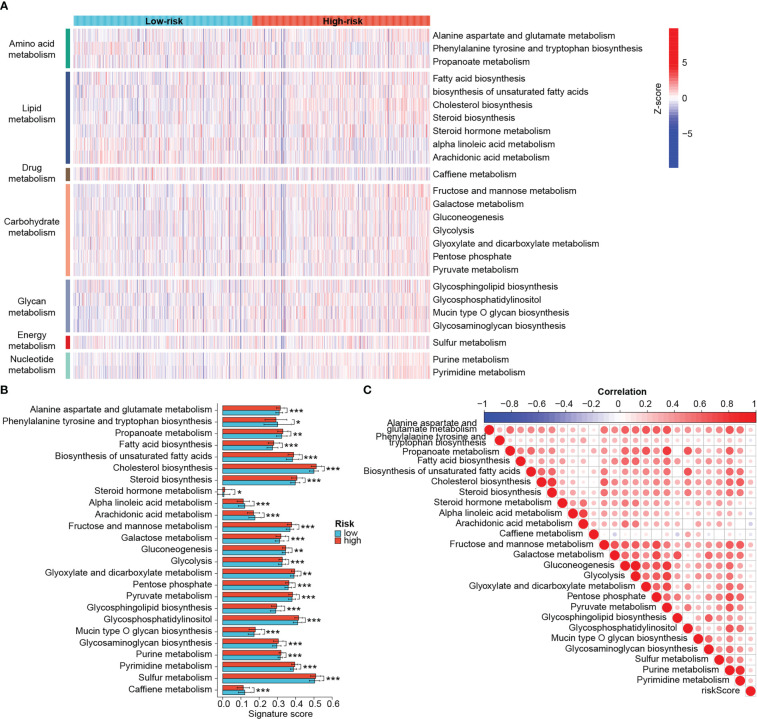
Distinct metabolic profiles between risk groups. **(A)** Heat map of different metabolic processes in risk groups. The color from blue to red represents the score from low to high. **(B)** Differential metabolic processes between risk groups. **(C)** Heat map of correlation between risk scores and metabolic processes. In this heat map, the size of the circle represents the statistical significance and the color represents the correlation. The color from blue to red represents the negative to positive correlation. (*P < 0.05, **P < 0.01, ***P < 0.001).

Metabolic reprogramming acts as an important driver of the tumoral adaptation to the microenvironment and can also influence the efficacy of antitumor therapy, so we sought to unravel the differences in metabolic status between the two groups. Metabolic pathways were mainly classified into those related to the metabolism of amino acids, lipids, carbohydrates, glycans, nucleotides, energy and drugs (**Figure 8A**). Epigenetic activation of the cholesterol synthesis is linked to treatment resistance and biological malignancy of BRCA (44). Targeting nucleotide metabolism, especially pyrimidine metabolism, is also considered promising for improving the efficacy of antitumor immunotherapy (45). As expected, there were differences in various metabolic pathways between groups, and the majority of them were significantly more active in the high-risk group, such as cholesterol biosynthesis, glycosaminoglycan biosynthesis, pyrimidine metabolism, etc. (**Figures 8A, B**). Higher scores of caffeine metabolism, alpha linoleic acid metabolism and arachidonic acid metabolism were found in the low-risk group (**Figure 8B**). According to the **Figure 8C**, the risk scores showed significant positive correlations with cholesterol synthesis, fructose and mannose metabolism, glycosaminoglycan biosynthesis and pyrimidine synthesis. Yet the negative association between arachidonic acid metabolism and risk score was relatively strong.

Furthermore, we also explored differences in cancer hallmark-related function between risk groups. High-risk tumors exhibited significantly higher heterogeneity and higher scores for proliferation, EMT, TGF-beta signaling, angiogenesis, m6A and exosome secretion than low-risk tumors (**Supplementary Figures S3A–C**). The apoptosis scores were elevated while the ferroptosis scores were decreased in low-risk patients (**Supplementary Figures S3A, B**). Moreover, the risk signature correlated well with those cancer hallmarks (**Supplementary Figure S3A**). These results show that high- and low-risk patients shared distinct tumor metabolic, proliferative, apoptotic, and metastatic characteristics, suggesting a good classifier effect of the prognostic signature.

### 3.8 Differences in DNA damage response and antitumor immunity

**Figure 9:**
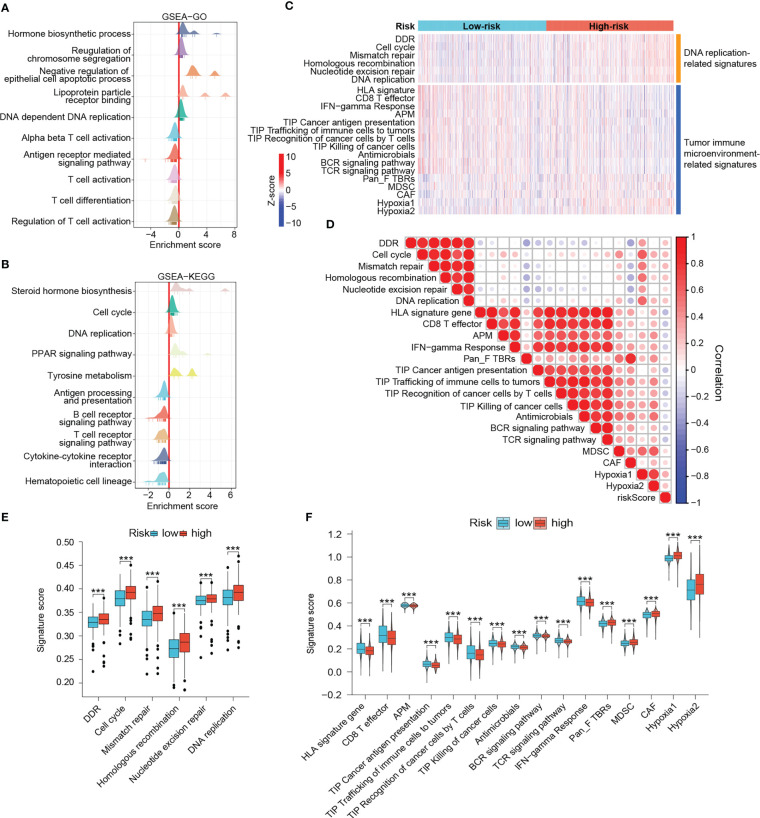
Outlining the DNA damage response and antitumor immunity landscapes. **(A, B)** GSEA results. In these ridge plots, an enrichment peak greater than 0 indicates that the pathway/term is enriched in the high-risk group while conversely, it is enriched in the low-risk group. **(C)** Heat map of DNA damage response and antitumor immunity signatures in risk groups. The color from blue to red represents the score from low to high. **(D)** Heat map of correlation between risk scores and different signatures. In this heat map, the size of the circle represents the statistical significance and the color represents the correlation. The color from blue to red represents the negative to positive correlation. Differential scores of DNA damage response **(E)** and antitumor immunity **(F)** between risk groups. (***P < 0.001).

Since the DRG-based signature has served as a good classifier in terms of patient prognosis, immune microenvironment, and metabolic activity, we wanted to explore in depth the functional differences between high- and low-risk groups. GSEA results demonstrated that immune response-related pathways such as antigen presentation and processing, T cell activation, B cell and T cell receptor signaling pathways, and cytokine - cytokine receptor interactions were significantly enriched in low-risk tumors, implying that low-risk tumors may be immune-enriched “hot tumor” phenotypes (**Figures 9A, B**). In contrast, hormone biosynthesis, cell cycle, and DNA replication pathways were noticeably enriched in the high-risk group (**Figures 9A, B**). The heat map demonstrated differences in DNA damage repair, antitumor immune response, and cancer hallmark-related scores among patients in different risk groups (**Figure 9C**). High-risk tumors possessed notably enhanced DNA damage response and DNA replication activities (**Figures 9C, E**). However, in terms of antitumor immunity, low-risk patients scored significantly higher in HLA signature, CD8^+^ effector T cells, tumor antigen presentation, and interferon-gamma response than those high-risk ones (**Figures 9C, F**). Moreover, the low-risk group dominated in four of the seven steps of cancer-immunity cycle (**Figures 9C, F**) (31, 46). In contrast, high-risk patients possessed markedly increased myeloid-derived suppressor cell (MDSC) scores, cancer-associated fibroblast (CAF) scores, and hypoxia scores (**Figures 9C, F**). Besides, risk scores were negatively associated with antitumor immunity-related signatures and positively associated with DNA replication, mismatch repair, CAF, MDSC and hypoxia-related signatures (**Figure 9D**). The microbiota has been reported to significantly contribute to the colonization of BRCA metastases and the efficiency of immunotherapy (47, 48). Interestingly, the anti-microbial scores were also notably higher in the low-risk group, which was in accordance with the aforementioned results of fewer tumor metastatic events and enhanced immunity in low-risk patients (**Figures 9C, F**). These results reinforce that low-risk patients experienced stronger antitumor immune responses and higher potential to benefit from immunotherapy.

### 3.9 The signature could distinguish potential responders for immunotherapy

**Figure 10:**
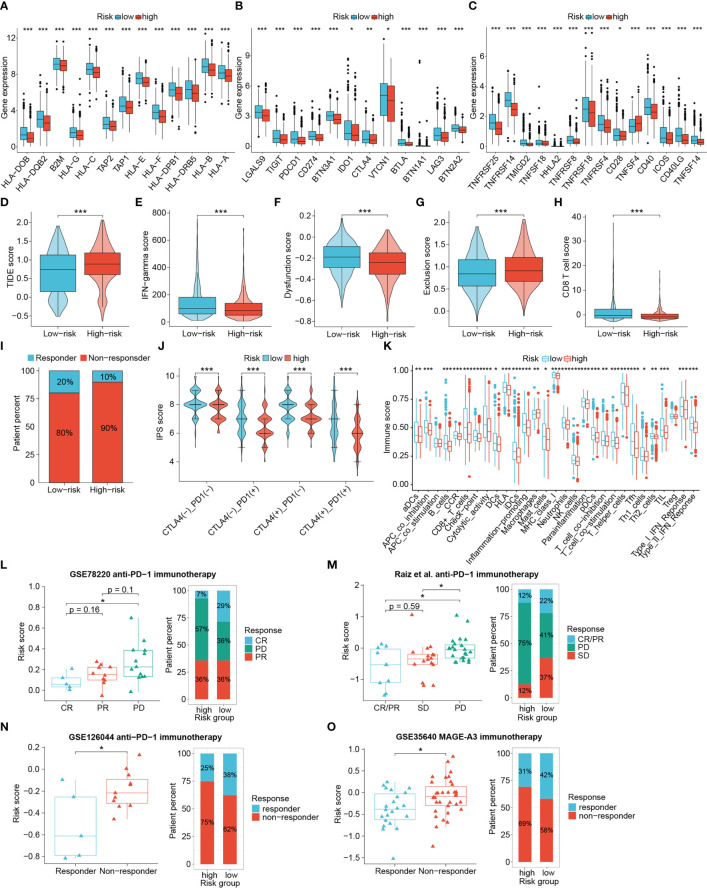
The value of the signature in differentiating immunotherapy responses of patients. The differential expression of HLA members **(A)**, immunoinhibitory molecules **(B)** and immunostimulatory molecules **(C)**. Differences in TIDE scores **(D)**, IFN-gamma scores **(E)**, T cell dysfunction scores **(F)**, T cell exclusion scores **(G)**, CD8+ T cell scores **(H)**, percentage of immunotherapy responders **(I)**, IPS scores **(J)** and immune function scores **(K)** between risk groups. **(L–O)** Four immunotherapy cohorts validated the predictive performance of the signature. (*P < 0.05, **P < 0.01, ***P < 0.001).

We next explored the practicality of the signature in signaling the responsiveness of immunotherapy recipients in a comprehensive manner by comparing the expression of key immunomodulatory molecules, TIDE scores, IPS scores, and other antitumor-immunity-related scores between different risk group. Subsequently, publicly available real-world immunotherapy cohorts validated the distinguishing efficacy of the signature. The expression of MHC complex-related molecules such as TAP1, TAP2, B2M and HLA genes as well as immune checkpoints including PDCD1, CTLA4, CD274, TIGIT, LAG3, CD40 and ICOS were elevated in low-risk tumors (**Figures 10A–C**). Low-risk tumors also exhibited higher interferon (IFN)-gamma scores, CD8^+^ T cell scores, and T-cell dysregulation scores, and lower TIDE scores and T-cell exclusion scores, indicating that low-risk patients had enhanced antitumor immunity, lower chances to experience immune evasion and improved responses to immunotherapy. (all *P* < 0.001) (**Figures 10D–H**). Responders to immunotherapy were also more distributed among low-risk patients (**Figure 10I**). IPS scores further confirmed the difference in susceptibility to immunotherapy among patients. **Figure 10J** showed that the low-risk patients achieved higher IPS scores than the high-risk patients in any CTLA4 and PD-L1 subgroups (*P* < 0.001). GSVA results also showed that scores for immune functional signatures including CD8^+^ T cells, cytolytic activity, MHC-I molecules, DC function and IFN response were enhanced in low-risk individuals (**Figure 10K**).

Given the important role of BRCA1/2 mutations in DNA damage and repair, we also analyzed the relevance of this signature to BRCA1/2 mutations. The BRCA1/2 mutation information in the TCGA-BRCA dataset was visualized using cBioPortal (www.cbioportal.org/) (**Supplementary Figure S4A**). We assessed whether the performance of the signature was stable by comparing the survival time between risk groups. In patients with BRCA1/2 mutation, the OS of low-risk patients remained longer than that of high-risk patients, but the difference was not statistically significant (*P* = 0.052, **Supplementary Figure S4B**). However, by comparing the TIDE scores we found that low-risk patients in the population harboring the BRCA1/2 mutation were still more favorable to benefit from immunotherapy (*P* < 0.05, **Supplementary Figure S4C**). These results demonstrate that low-risk patients were considered more suggestive as responders to immunotherapy.

To further check the proficiency of this signature in predicting immunotherapeutic benefits of patients, here we introduced four publicly available real-world immunotherapy cohorts to validate the above results. Analyses of three cohorts receiving anti-PD-1 therapy revealed that patients with better therapeutic outcomes attained lower risk scores, and a higher proportion of responders were found in the low-risk group (**Figures 10L–N**). In addition, low-risk patients were also more responsive to MAGE-A3 immunotherapy (**Figure 10O**). Accordingly, the DRG-based risk signature could distinguish patient sensitivity to immunotherapy, with low-risk patients being more likely to achieve favorable responses and improved therapeutic outcomes.

### 3.10 Chemotherapeutic sensitivity assessment and screening for optimal small molecule agents

**Figure 11:**
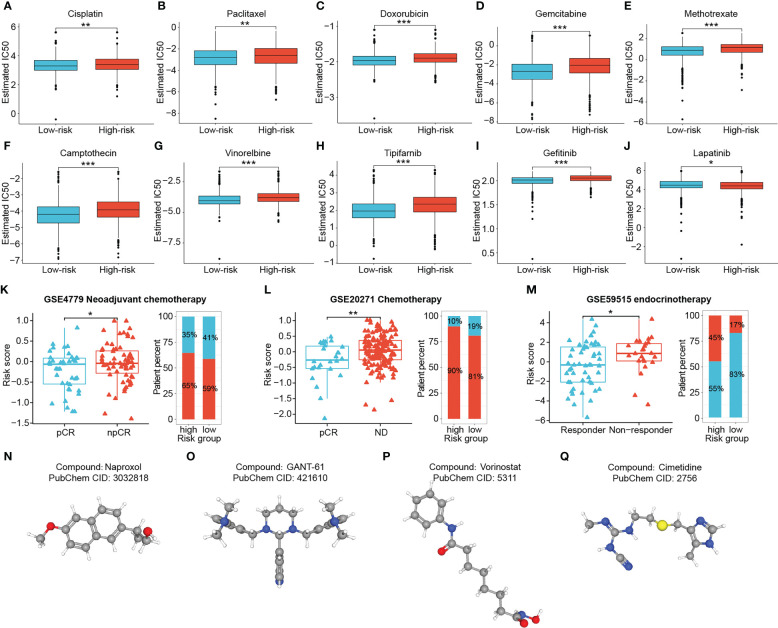
Chemotherapy sensitivity prediction and candidate drug exploration. Low-risk patients presented higher sensitivity to Cisplatin **(A)**, Paclitaxel **(B)**, Doxorubicin **(C)**,Gemcitabine **(D)**, Methotrexate **(E)**, Camptothecin **(F)**, Vinorelbine **(G)**, Tipifarnib **(H)** and Gefitinib **(I)**, and lower sensitivity to Lapatinib **(J)**. **(K–M)** Three cohorts receiving neoadjuvant chemotherapy, chemotherapy, and neoadjuvant endocrinotherapy validated the predictive performance of the signature. **(N–Q)** (*P < 0.05, **P < 0.01, ***P < 0.001). (ns: not significant, *P < 0.05, **P < 0.01, ***P < 0.001).

To augment the utility of this signature for advising clinical individualized regimens for BRCA, we applied the “pRRophetic” package to predict the chemotherapy sensitivity of patients, and validated them in combination with three real-world cohorts. The IC50 values for the majority of clinical conventional chemotherapeutic agents (Cisplatin, Paclitaxel, Doxorubicin, Gemcitabine, Methotrexate, Camptothecin and Vinorelbine) and targeted agents (Tipifarnib and Gefitinib) were remarkably lower in low-risk patients, suggesting that they were more sensitive to these drugs (all *P* < 0.01) (**Figures 11A–I**). While the high-risk group showed better sensitivity to Lapatinib (*P* < 0.05) (**Figure 11J**). Moreover, risk scores exhibited significant positive correlations with the IC50 for the first nine drugs, with the most obvious correlations with Gemcitabine and Gefitinib (all *P* < 0.001) (**Supplementary Figure S5**). Validation using two cohorts receiving neoadjuvant chemotherapy with Fluorouracil, Doxorubicin and Cyclophosphamide showed significantly lower risk scores in the responder group than in the non-responder group (all *P* < 0.05) (**Figures 11K, L**). Interestingly, the difference in risk scores was still observed between the responder and non-responder groups of the Letrozole-based endocrinotherapy cohort (*P* < 0.05) (**Figure 11M**).

Considering that high-risk patients suffered from poor outcomes and potential resistance to multiple therapeutic regimens, we screened for drug candidates suitable for high-risk patients by matching the expression profile variations. Differential genes between two risk groups were analyzed and uploaded to the Cmap database. Four potentially effective small molecule compounds for high-risk tumors were predicted, namely Naproxol (**Figure 11N**), GANT-61 (**Figure 11O**), Vorinostat (**Figure 11P**) and Cimetidine (**Figure 11Q**) (**Supplementary Table S4**).

### 3.11 Experimental verification of the expression and functional characteristics of key DRGs

**Figure 12:**
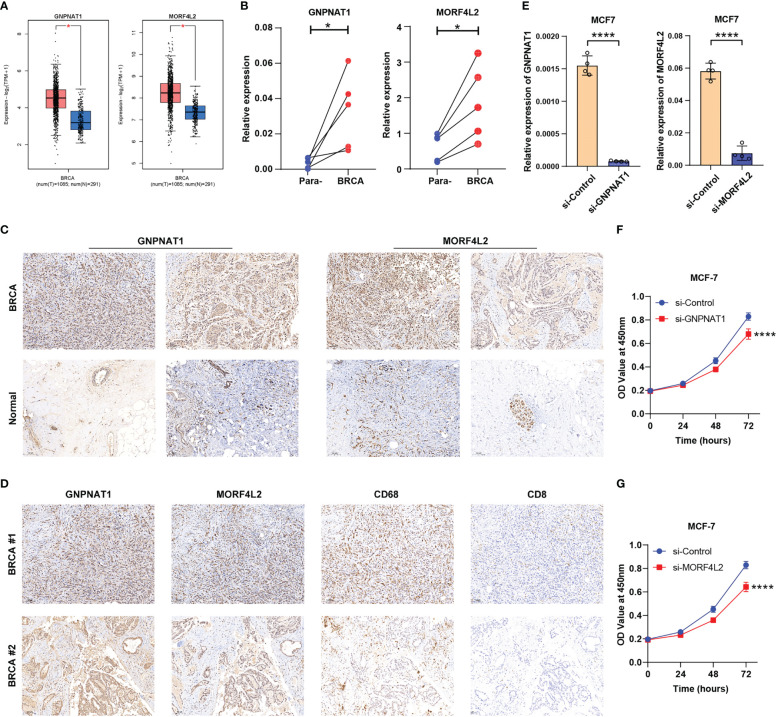
Expression validation and functional exploration of two key DRGs. **(A)** GEPIA database analyses demonstrated the differential expression of GNPNAT1 and MORF4L2 in BRCA and normal breast tissues. **(B, C)** RT-qPCR and IHC (10×) experiments showed the elevated expression of GNPNAT1 and MORF4L2 in BRCA tissues compared with adjacent normal tissues. **(D)** IHC assays (10×) revealed the association of key DRGs with macrophages and CD8^+^ T cells. **(E)** The expression of GNPNAT1 and MORF4L2 was knocked down in the targeted group. **(F, G)** Knockdown of either GNPNAT1 or MORF4L2 could suppress the proliferation activity of MCF-7 cells. (*P < 0.05, ****P < 0.0001).

After a comprehensive evaluation of the DDR-based signature, we aimed to further validate the expression and biological functions of key DRGs in BRCA. Two DRGs, GNPNAT1 and MORF4L2, exhibited strong correlations with survival prognosis and tumor-infiltrating immune cells (CD8^+^ T cells, macrophages, etc.) in the analysis above (**Figure 7C** and **Supplementary Table S3**). Although GNPNAT1 has been reported to be associated with radiotherapy sensitivity in BRCA, the majority of roles of these two DRGs in BRCA have not been adequately characterized, resulting in their focus by us. Through survival analyses based on multiple external datasets, the association of high expression of these two DRGs with shorter OS, RFS, MFS, etc. was further revealed (**Supplementary Figure S6**). Elevated expression of GNPNAT1 and MORF4L2 in BRCA was verified by GEPIA database (http://gepia.cancer-pku.cn/) and our qRT-PCR results of clinical samples (**Figures 12A, B**). IHC sections also showed higher protein level of GNPNAT1 and MORF4L2 in BRCA tissues (**Figure 12C**). Furthermore, consistent with the findings described above, we found significant co-localization of the positive stained regions of GNPNAT1 and MORF4L2 with the marker of macrophages CD68 (**Figure 12D**). However, few CD8^+^ T cells were detected within these regions (**Figure 12D**). Since high levels of GNPNAT1 and MORF4L2 expression suggest poor prognosis, yet whether they affect the proliferation of BRCA cells is unclear, so we investigated their effect on the proliferation of MCF-7 cells. The designed siRNAs of GNPNAT1 and MORF4L2 both possessed favorable gene silencing efficiency (**Figure 12E**). CCK-8 assays revealed that knockdown of both DRGs could result in impaired proliferation viability of MCF-7 cells (**Figures 12F, G**).

## 4 Discussion

The DNA damage repair (DDR) machinery is an essential part for the cellular maintenance of genomic stability, and its function deficiency or abnormality could induce alterations in basic biological behaviors such as cell proliferation and apoptosis (6). In breast cancer (BRCA), the most serious threat in cancer-related deaths among women, the role of DDR machinery cannot be neglected since it was proposed to be closely involved in the initiation, progression, metastasis, recurrence, and treatment resistance of tumors (6, 8, 9). For example, loss of BRCA1 or its two partners NUMB and HES1 in the mammary epithelium triggered DDR defects and promoted basal/mesenchymal transdifferentiation and tumorigenesis (49). The presence of RAD51 foci was reported to be a promising biomarker indicating the poly (ADP-ribose) polymerase (PARP) inhibitor (PARPi) sensitivity in germline BRCA (50). High level of ATM protein, one of the important kinases involved in DDR signaling, was found to correlate with the recurrence of BRCA (51). Accordingly, it is relevant to fully explore and identify DDR-related molecules with prognostic and therapeutic value in BRCA.

Immunotherapy is believed to harbor great potential for clinical application given its ability to activate the immune cells of the body and reboot effective antitumor immunity. However, currently immunotherapy suffers from a limited responsive population, and several factors such as TME, metabolic reprogramming and genomic instability affect the efficacy of immunotherapy (18, 52, 53). Hence, a comprehensive and multidimensional characterization of BRCA patients is necessary. Notably, although risk signature of BRCA based on DNA damage- or repair-related genes with good prognostic effects have been established previously, signatures enabling comprehensive analyses of patient prognosis, TME, tumor metabolic profiles, and immunotherapy and chemotherapy sensitivity in an integrated multi-omics manner are still lacking (24, 54, 55). Accordingly, we developed a prognostic signature based on 27 DRGs and further comprehensively characterized patients in terms of prognosis, immune microenvironment, antitumor immunity and tumor metabolism, etc. Moreover, the long-term prognostic efficacy and the generalizability to multiple external cohort of this signature was also been focused on, as well as the mining of key molecules and effective drugs.

The survival outcomes and clinicopathological features of patients with different risk are a direct reflection of the prognostic efficacy. The significantly distinct survival outcomes of high-risk patients were demonstrated in multiple cohorts, survival types, molecular subtypes, and pathological subgroups. Besides, risk stratification by the model correlated well with clinical factors such as survival outcomes, tumor stage, progressive events, and tumor size of BRCA patients. Therefore, we wanted to learn more about the 27 DDR molecules comprising this robust signature. After reviewing the existing literature, we found that the cancer-promoting or cancer-inhibiting function of some of the 27 DRGs in oncology has been reported. For example, MECOM (MDS1 and EVI1 complex locus), was reported to be engaged in modulating the cancer-stem-cell (CSC) properties in lung squamous carcinoma (56). Replication protein A3 (RPA3) inhibited protective autophagy and promoted cisplatin resistance in lung adenocarcinoma (57). Activation of nuclear factor of activated T cells 2 (NFATC2) could alleviate the functional exhaustion of tumor-infiltrating CD8^+^ T cells (58). The unfavorable prognostic effects of S100A11 (S100 calcium binding protein A11) were reflected by pro-inflammation and pro-fibrosis, and the facilitation of cancer cell proliferation and migration in hepatocellular carcinoma models (59). Moreover, the oncogenic role of WNT7B, the Wnt family member 7B, has been reported in breast cancer previously, with a major focus on its promotion of invasive behaviors such as angiogenesis and metastasis (60, 61).

Since the effects of some DRGs in regulating tumor behaviors have been described, and their cancer-promoting or -suppressing roles are broadly consistent with the risk of DRGs in this study, it also reinforces the significance of this study. Therefore, we further identified six DRGs associated with tumor progression by clinicopathological relevance analysis. Actin like 6A (ACTL6A) showed higher level in tumors with advanced stage, and it has been recognized for its contribution to tumorigenesis, proliferation and invasion, and it also promotes the repair of DNA damage induced by cisplatin in tumor cells (62–64). The present study found that high expression of mortality factor 4 Like 2 (MORF4L2) was associated with worse clinical features, more infiltrated macrophages, and stronger BRCA proliferative activity, as reported for the first time so far. Its value in assessing 5-Fluorouracil (5-FU) resistance and disease progression of tumors has been revealed (65, 66). DCTP Pyrophosphatase 1 (DCTPP1) was also reported to potentiate BRCA proliferation through DNA-damage-repair pathway (67). The oncological role of TANK (TRAF family member-associated NF-κB activator) was rarely reported, but its known effect in antiviral innate immunity as well as interaction with immunomodulatory molecules such as TRAF family proteins and TBK1 makes it a promising target (18, 68). High glucosamine-phosphate N-acetyltransferase 1 (GNPNAT1) expression predicted poor prognosis in lung cancer and breast cancer, but was also observed in radiotherapy-sensitive breast cancer cells (69–71). We first uncovered that knockdown of GNPNAT1 inhibited the proliferation of human BRCA cells. VAV3, Vav guanine nucleotide exchange factor 3, an independent prognostic factor in BRCA, mediated lung metastasis and endocrine therapy resistance of BRCA (72, 73). In the present study, apart from the identifying the relationship of these DRGs with tumor progression, their association with tumor-infiltrating immune cells was also revealed.

The contribution of diverse infiltrative immune cells in TME to survival prognosis and therapeutic resistance of patients cannot be underestimated, especially for cancer immunotherapy (13). There were more infiltrating CD8^+^ T cells, follicular helper T cells, gamma delta T cells, and M1 macrophages, and fewer M0 macrophages and M2 macrophages inside the low-risk tumors. In most cancers, including BRCA, the intratumoral abundance of CD8^+^ cytotoxic T lymphocytes, major antitumor effector cells within the TME, is a favorable prognostic marker. Follicular helper T cells participated in the regulation of B-cell immunity, and their crosstalk with CD8^+^ T cells is required for the efficacy of immunotherapy (74, 75). M1 macrophages, in addition to possessing tumor-killing effects themselves, could also augment the cytotoxic effects of CD8^+^ T cells by attracting more T lymphocytes (76). While the M0 and M2 phenotypes of tumor-associated macrophages (TAMs) are generally correlated with poor prognosis (77). M2 macrophages are important immunosuppressive TAMs with the capability to promote cancer invasion and metastasis and to constrain the function of CD8^+^ T cells and immunotherapeutic efficacy (78). Furthermore, high-risk patients also presented elevated CAF scores and MDSC scores. CAFs and MDSCs also function to reshape the immunosuppressive microenvironment and pave for the development of malignant progression and therapeutic resistance of tumors (79, 80). Thus, tumor-infiltrating immune cell characteristics in different risk groups enable a favorable link with patient prognosis and response to immunotherapy.

Metabolic reprogramming of cancer cells is also one of the crucial mechanisms for their adaptation to the survival environment (53). Therefore, an integrated analysis of the immune microenvironment and tumor metabolism is necessary. Lipid and cholesterol anabolism is elevated in multiple highly proliferative cancer cells and is often associated with tumor malignant invasiveness and treatment resistance, especially to endocrine therapy (44). Furthermore, the excessive accumulation of lipids and lactate typically shapes the immunosuppressive microenvironment and aggravates immune evasion (44, 53). The upregulated pyrimidine metabolism and microenvironmental pyrimidine metabolites have been found to diminish the tumor-killing effect of chemotherapeutic agents (45, 81). These findings support the features of high lipid metabolism scores, immunosuppression status, and poor prognosis and treatment responsiveness of the high-risk individuals in the present analysis.

Combining the infiltrative immune cell abundance, tumor metabolic profile, immune function score, checkpoint expression, and immunotherapy prediction algorithms including TIDE and IPS, we derived the finding that low-risk patients stratified by this DNA damage-repair-related signature could respond better to immunotherapy. Additionally, low-risk patients also experienced improved response to various traditional chemotherapeutic agents and targeted agents. Six real-world cohorts, four receiving ICB or MAGE-A3 therapy, two receiving combination chemotherapy, further confirmed these predictive results. Notably, enhanced lipid and cholesterol metabolism was reported to be associated with endocrinotherapy resistance (44). And results of metabolic scores and real-world cohort validation are compatible with that.

Despite the fact that high-risk patients may experience relatively limited responsiveness to chemotherapy and immunotherapy, we screened four promising agents, Naproxol, GANT-61, Vorinostat, and Cimetidine. Among them, GANT-61 was described to inhibit the invasion and metastasis of TNBC in combination with a nano-delivery system (82). Naproxol belongs to non-steroidal anti-inflammatory drugs, and it was reported that hyaluronic acid-encapsulated naproxen could target BRCA stem cells in a Cyclooxygenase (COX) non-dependent manner (83). As the compound capable of modulating receptor status, inducing apoptosis, and inhibiting EMT in BRCA, Vorinostat has been used in combination with chemotherapeutic agents, antiestrogenic drugs, monoclonal antibodies, and nano-delivery systems in several studies (84–86). Cimetidine was also found to exhibit anti-neoplastic properties inhibiting the growth of BRCA cells (87).

Although we have endeavored to present a comprehensive characterization of prognosis, immune microenvironment and immune function, tumor metabolism, and chemotherapy and immunotherapy sensitivity of BRCA patients based on a molecular signature consisting of 27 DRGs, and to provide alternatives for high-risk patients with underlying resistance to conventional therapeutic regimens, we are well aware that this study does have some shortcomings. Firstly, the functions of some of the signature DRGs have not been fully elucidated and need to be further clarified in subsequent studies. Secondly, the prognostic efficacy of the signature remains to be tested in a larger cohort of local patients. Finally, predictive models based on RNA-seq data still face the problem of limited usage and available data in clinical practice.

## 5 Conclusion

In summary, a prognostic signature based on 27 DRGs was developed, which exhibited great performance in indicating prognosis, immune microenvironment and immune function profiles, tumor metabolic status, and therapeutic sensitivity of BRCA patients. Candidate compounds were provided for patients with underlying therapeutic resistance. Preliminary experiments verified the expression characteristics as well as biological functions of key DRGs, GNPNAT1 and MORF4L2. This study may contribute new perspectives for precision medicine and molecular target research of BRCA.

## Data availability statement

The original contributions presented in the study are included in the article/**Supplementary Material**. Further inquiries can be directed to the corresponding authors. Codes have been uploaded in the online depository (https://github.com/will2023204/Code.git). Further reasonable inquiries can be directed to the corresponding author.

## Ethics statement

The studies involving human participants were reviewed and approved by Ethics Committee of School of Basic Medicine, Naval Medical University. The patients/participants provided their written informed consent to participate in this study.

## Author contributions

CL and SYu contributed equally to this work, performed the experiments, and wrote the manuscript. CL and SYu conducted the bioinformatic analyses. JC, SW and QH helped with the data collection and arrangement. CQ and SYu designed the study and offered guidance. All authors contributed to the article and approved the submitted version.

## Supplementary material

Supplementary Figure 1 Validation of the prognostic performance of the signature in various clinicopathological subgroups in cohorts TCGA-BRCA **(A)**, GSE96058 **(B)** and GSE20685 **(C)**.

Supplementary Figure 2 Cox regressions identified the DNA damage repair-related signature capable of acting as an independent prognostic indicator for PFS, DFS and DSS in TCGA-BRCA cohort.

Supplementary Figure 3 Differences in cancer hallmark-related scores between risk groups. **(A)** Heat map of correlation between risk scores and hallmark signatures. The color from blue to red represents the score from low to high. **(B, C)** Differential hallmark-related signature scores between risk groups. (ns: not significant, **P* < 0.05, ***P* < 0.01, ****P* < 0.001).

Supplementary Figure 4 Association between the risk signature and BRCA1/2 mutations. **(A)** Frequency and pattern of BRCA1/2 mutations in BRCA patients in the TCGA cohort. **(B)** Survival differences between the high- and low-risk patients in different BRCA1/2 mutation subgroups. **(C)** Differences in TIDE scores between the high- and low-risk patients with wild-type BRCA1/2 or mutant BRCA1/2. (**P* < 0.05, ***P* < 0.01, ****P* < 0.001).

Supplementary Figure 5 Correlation between risk scores and IC50 values of the 10 chemotherapeutic drugs mentioned above.

Supplementary Figure 6 Analysis of external datasets demonstrated poor prognosis of BRCA patients with high expression of GNPNAT1 **(A)** or MORF4L2 **(B)**.
