# Supplementary figures and images for: Risk stratification based on DNA damage-repair-related signature reflects the microenvironmental feature, metabolic status and therapeutic response of breast cancer
Source: Front Immunol. 2023 Mar 24;14:1127982. doi: 10.3389/fimmu.2023.1127982 (PMC10080010; doi:10.3389/fimmu.2023.1127982)

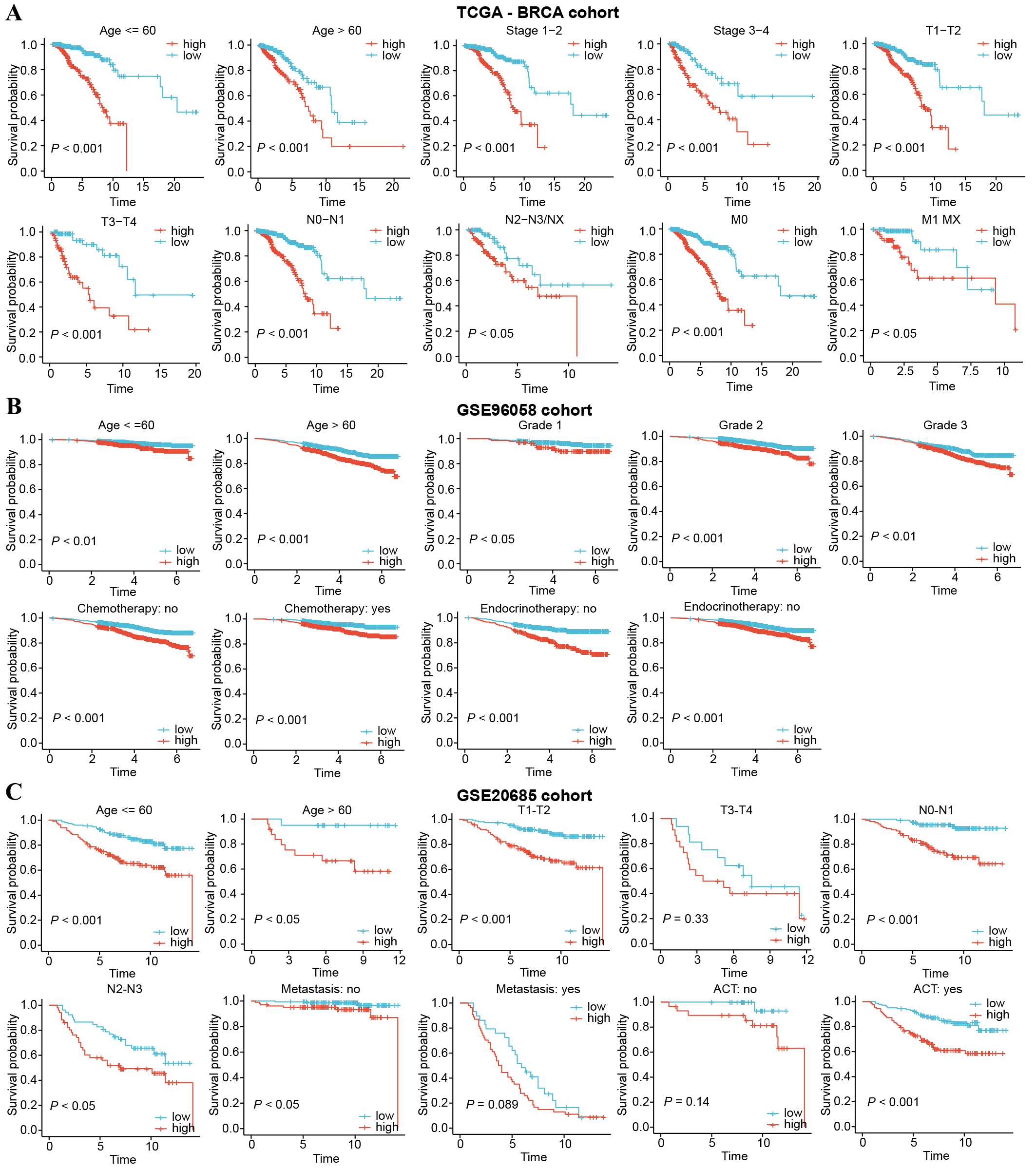

Supplement: Supplementary Figure 1 — Validation of the prognostic performance of the signature in various clinicopathological subgroups in cohorts TCGA-BRCA (A), GSE96058 (B) and GSE20685 (C). [file Image_1.tif]

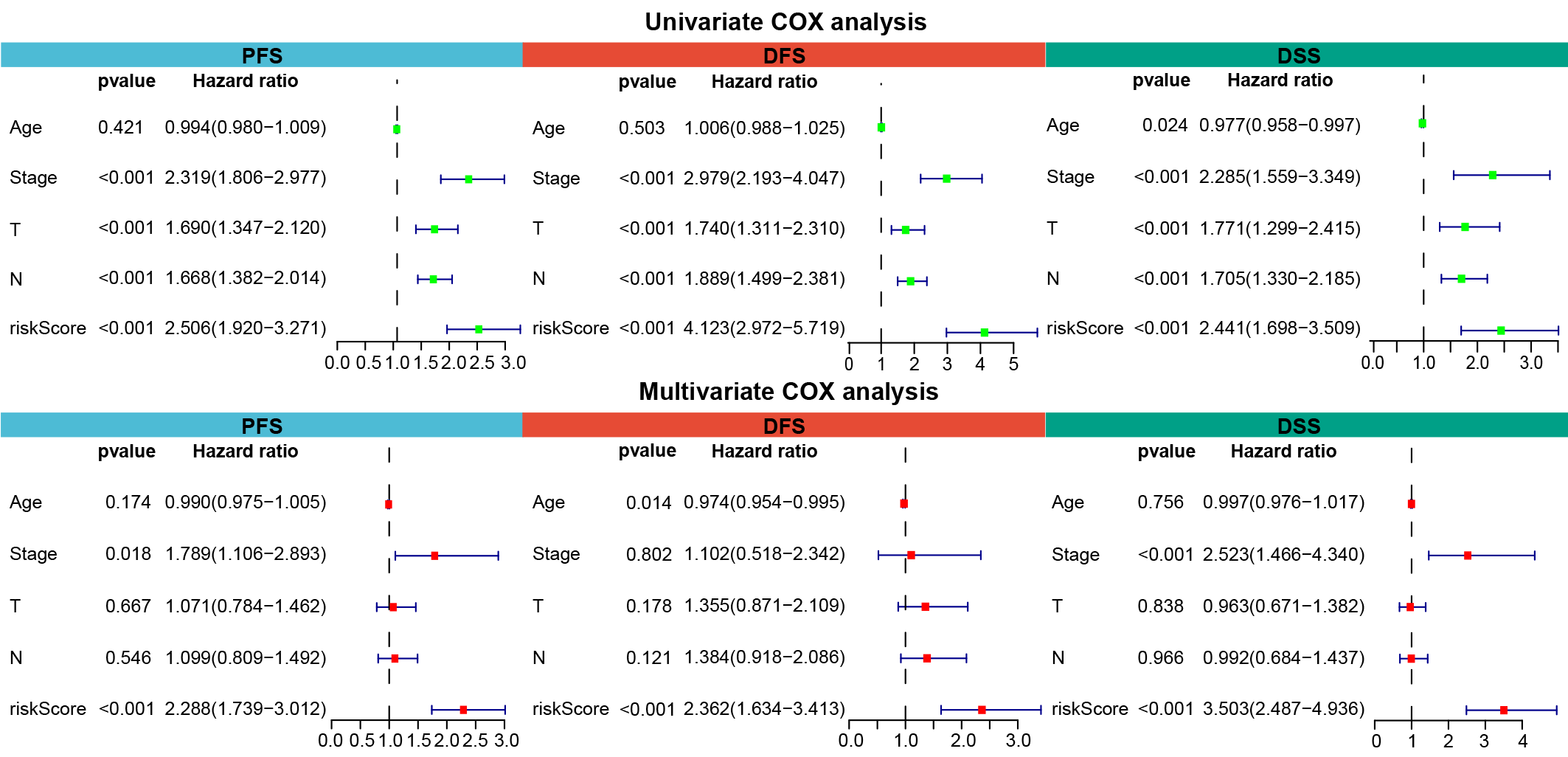

Supplement: Supplementary Figure 2 — Cox regressions identified the DNA damage repair-related signature capable of acting as an independent prognostic indicator for PFS, DFS and DSS in TCGA-BRCA cohort. [file Image_2.tif]

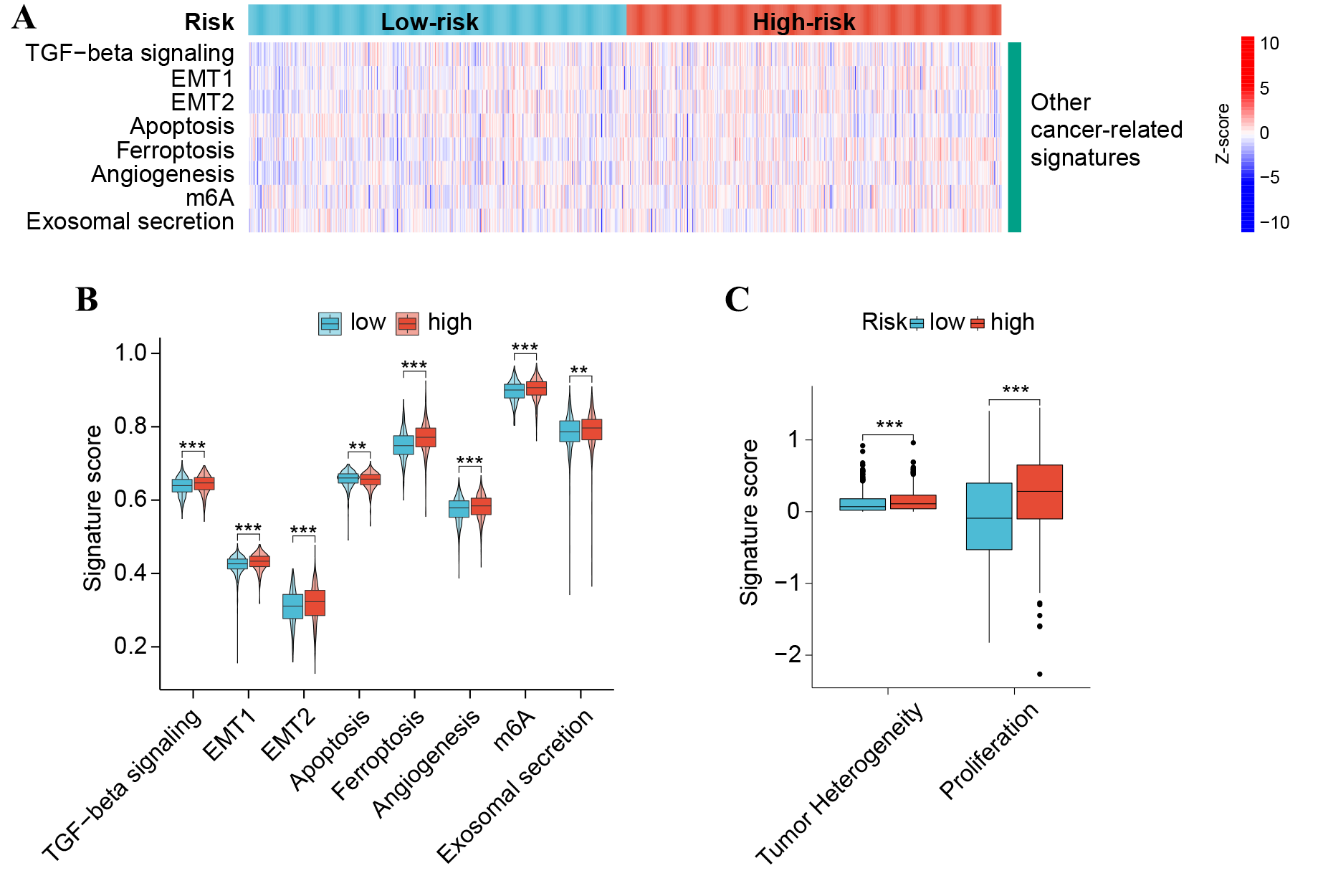

Supplement: Supplementary Figure 3 — Differences in cancer hallmark-related scores between risk groups. (A) Heat map of correlation between risk scores and hallmark signatures. The color from blue to red represents the score from low to high. (B, C) Differential hallmark-related signature scores between risk groups. (ns: not significant, *P < 0.05, **P < 0.01, ***P < 0.001). [file Image_3.tif]

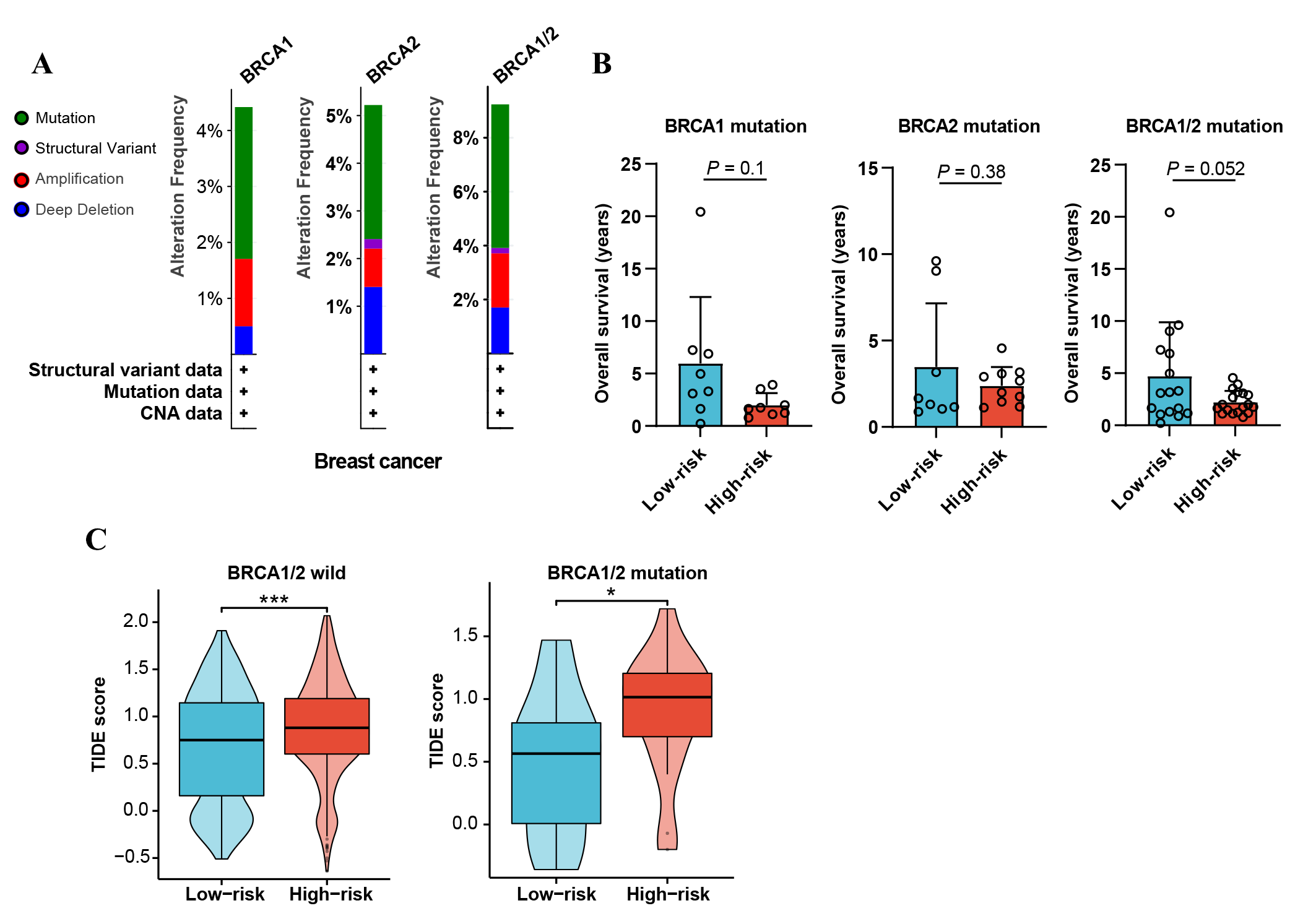

Supplement: Supplementary Figure 4 — Association between the risk signature and BRCA1/2 mutations. (A) Frequency and pattern of BRCA1/2 mutations in BRCA patients in the TCGA cohort. (B) Survival differences between the high- and low-risk patients in different BRCA1/2 mutation subgroups. (C) Differences in TIDE scores between the high- and low-risk patients with wild-type BRCA1/2 or mutant BRCA1/2. (*P < 0.05, **P < 0.01, ***P < 0.001). [file Image_4.tif]

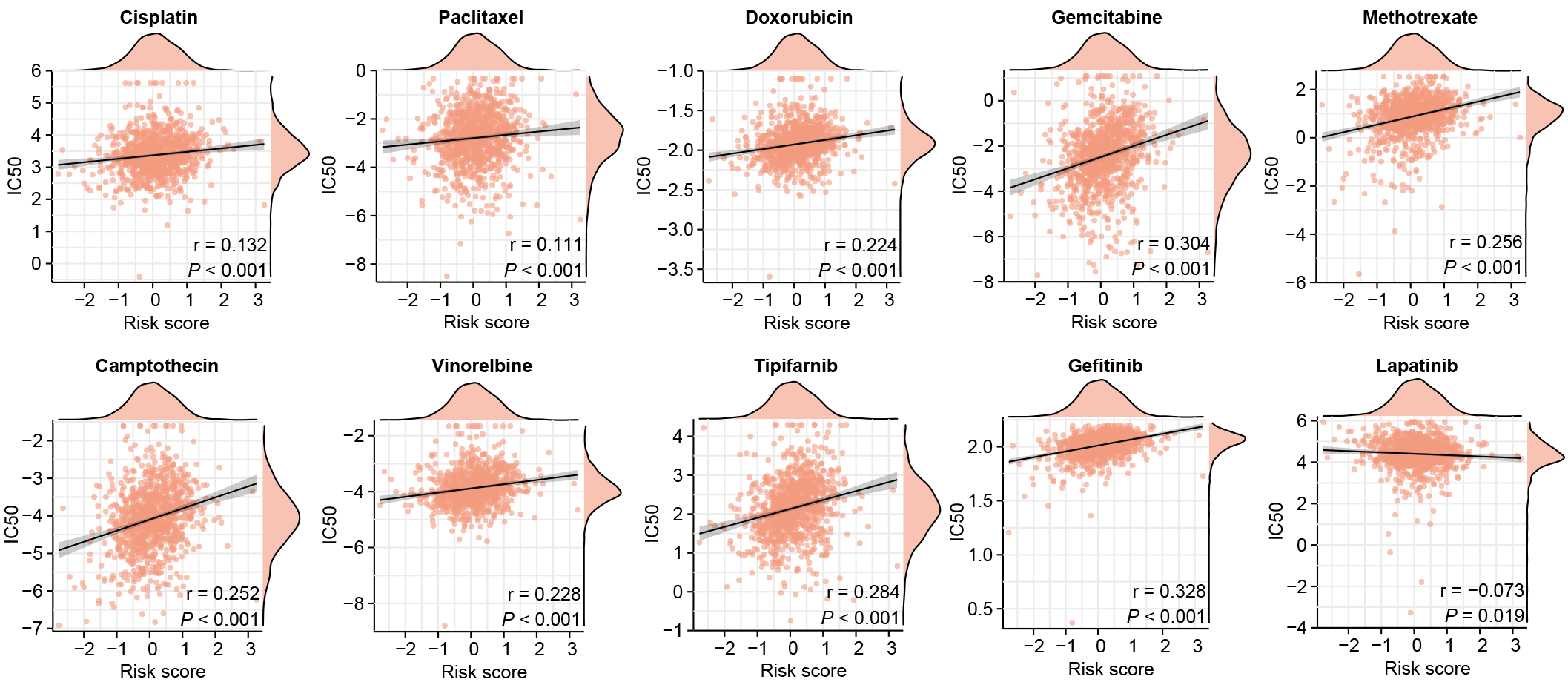

Supplement: Supplementary Figure 5 — Correlation between risk scores and IC50 values of the 10 chemotherapeutic drugs mentioned above. [file Image_5.tif]

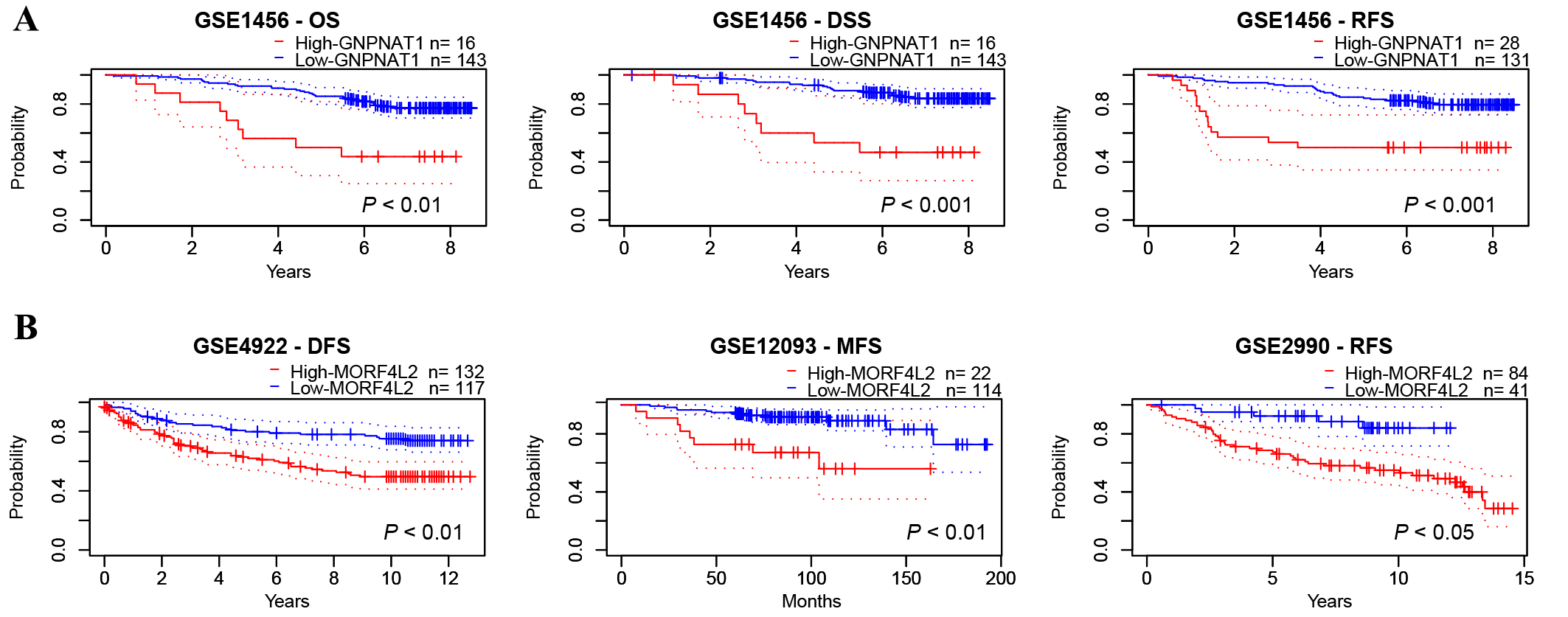

Supplement: Supplementary Figure 6 — Analysis of external datasets demonstrated poor prognosis of BRCA patients with high expression of GNPNAT1 (A) or MORF4L2 (B). [file Image_6.tif]
