# Supplementary material for: Risk stratification based on DNA damage-repair-related signature reflects the microenvironmental feature, metabolic status and therapeutic response of breast cancer
Source: Front Immunol. 2023 Mar 24;14:1127982. doi: 10.3389/fimmu.2023.1127982 (PMC10080010; doi:10.3389/fimmu.2023.1127982)
Supplement: Supplementary file 7 [file DataSheet_1.docx]

**SUPPLEMENTARY MATERIALS**

**Supplementary Table S1 | Clinical characteristics of the patients in the BRCA cohorts analyzed in this study**

| **Characteristics** | **TCGA-BRCA** | **GSE20685** | **GSE96058** |
| --- | --- | --- | --- |
| **Number of samples enrolled** | 1023 | 327 | 3273 |
| **Age** | - | - | - |
| <=60 | 573 (56.0%) | 282 (86.2%) | 1314 (40.1%) |
| >60 | 450 (44.0%) | 45 (13.8%) | 1959 (59.9%) |
| **PAM50 subtype** | - | - | - |
| Luminal A | 473 (46.2%) | NA | 1657 (50.6%) |
| Luminal B | 182 (17.8%) | NA | 729 (22.3%) |
| Her2 | 67 (6.5%) | NA | 327 (10.0%) |
| Normal | 34 (3.3%) | NA | 221 (6.8%) |
| Basal | 157 (15.3%) | NA | 339 (10.4%) |
| unknown | 110 (10.8%) | NA | 0 (0.0%) |
| **AJCC stage** | - | - | - |
| Stage 1 | 177 (17.3%) | NA | NA |
| Stage 2 | 578 (56.5%) | NA | NA |
| Stage 3 | 227 (22.2%) | NA | NA |
| Stage 4 | 19 (1.9%) | NA | NA |
| Unknown | 22 (2.2%) | NA | NA |
| **Nottingham histological grade (NHG)** | - | - | - |
| Grade 1 | NA | NA | 496 (15.2%) |
| Grade 2 | NA | NA | 1532 (46.8%) |
| Grade 3 | NA | NA | 1184 (36.2%) |
| Unknown | NA | NA | 61 (1.9%) |
| **T stage** | - | - | - |
| T1 | 274 (26.8%) | 101 (30.9%) | NA |
| T2 | 584 (57.1%) | 188 (57.5%) | NA |
| T3 | 126 (12.3%) | 26 (8.0%) | NA |
| T4 | 36 (3.5%) | 12 (3.7%) | NA |
| unknown | 3 (0.3%) | 0 (0.0%) | NA |
| **N stage** | - | - | - |
| N0 | 479 (46.8%) | 137 (41.9%) | NA |
| N1 | 347 (33.9%) | 87 (26.6%) | NA |
| N2 | 109 (10.7%) | 63 (19.3%) | NA |
| N3 | 71 (6.9%) | 40 (12.2%) | NA |
| unknown | 17 (1.7%) | 0 (0.0%) | NA |
| **M stage** | - | - | - |
| M0 | 849 (83.0%) | 244 (74.6%) | NA |
| M1 | 21 (2.1%) | 83 (25.4%) | NA |
| unknown | 153 (15.0%) | 0 (0.0%) | NA |
| **Survival status** | - | - | - |
| Alive | 877 (85.7%) | 244 (74.6%) | 2937 (89.7%) |
| Dead | 146 (14.3%) | 83 (25.4%) | 336 (10.3%) |
| **Survival time** | - | - | - |
| Median months (range) | 30.6  (2.1-286.8) | 98.57  (4.9-171.6) | 30.6  (1.9-80.3) |

**Supplementary Table S2 | Primers and siRNA sequences**

| **Species** | **Name** | **Application** | **Sequence** |
| --- | --- | --- | --- |
| Human | GNPNAT1-F | Primer for qRT-PCR | ACTCCTATGTTTGACCCAAGTCT |
| Human | GNPNAT1-R | Primer for qRT-PCR | TCTGTTAGCTGACCCAATACCT |
| Human | MORF4L2-F | Primer for qRT-PCR | GCAGGGTTCTCAACCTCGTG |
| Human | MORF4L2-R | Primer for qRT-PCR | TGCATGTTGCTTCTAGTTGGTTT |
| Human | GAPDH-F | Primer for qRT-PCR | GGAGCGAGATCCCTCCAAAAT |
| Human | GAPDH-R | Primer for qRT-PCR | GGCTGTTGTCATACTTCTCATGG |
| Human | si-GNPNAT1-sense | siRNA interference | CCUUGAAUGUCUACCACAATT |
| Human | si-MORF4L2-sense | siRNA interference | GAGGCGUUUAAGAAUAGAATT |

**Supplementary Table S3 | A total of 51 prognostic DRGs**

| **No.** | **Gene Symbol** | **HR (95% CI)** | ***P*.value** |
| --- | --- | --- | --- |
| 1 | MECOM | 1.139 (1.019-1.273) | 0.022 |
| 2 | RPA3 | 1.061 (1.012-1.112) | 0.013 |
| 3 | MORF4L2 | 1.002 (1.000-1.004) | 0.048 |
| 4 | PLK1 | 1.028 (1.003-1.053) | 0.029 |
| 5 | ACTL6A | 1.014 (1.000-1.029) | 0.050 |
| 6 | NFKB2 | 0.964 (0.938-0.992) | 0.011 |
| 7 | NFATC2 | 0.867 (0.778-0.966) | 0.010 |
| 8 | S100A11 | 1.000 (1.000-1.001) | 0.022 |
| 9 | VDAC3 | 1.005 (1.001-1.009) | 0.021 |
| 10 | FN1 | 1.001 (1.000-1.001) | 0.042 |
| 11 | PSMB8 | 0.990 (0.983-0.997) | 0.007 |
| 12 | PSMD14 | 1.053 (1.004-1.103) | 0.032 |
| 13 | TANK | 0.915 (0.861-0.972) | 0.004 |
| 14 | FBXO6 | 0.962 (0.930-0.995) | 0.024 |
| 15 | TNIP1 | 0.954 (0.929-0.980) | 0.001 |
| 16 | WNT7B | 1.053 (1.017-1.089) | 0.003 |
| 17 | FOS | 0.998 (0.996-1.000) | 0.046 |
| 18 | TONSL | 1.059 (1.004-1.117) | 0.035 |
| 19 | PSMD2 | 1.009 (1.001-1.017) | 0.027 |
| 20 | HLA-DQB1 | 0.990 (0.982-0.998) | 0.014 |
| 21 | HLA-DQA1 | 0.985 (0.975-0.996) | 0.007 |
| 22 | VAV3 | 0.991 (0.982-0.999) | 0.033 |
| 23 | PLAU | 1.006 (1.002-1.010) | 0.004 |
| 24 | GNPNAT1 | 1.025 (1.006-1.044) | 0.008 |
| 25 | PSME1 | 0.994 (0.990-0.998) | 0.001 |
| 26 | JUN | 0.996 (0.992-1.000) | 0.044 |
| 27 | JUNB | 0.997 (0.994-1.000) | 0.025 |
| 28 | LEF1 | 0.962 (0.928-0.997) | 0.034 |
| 29 | INPP1 | 0.871 (0.769-0.986) | 0.029 |
| 30 | POLR2K | 1.009 (1.001-1.017) | 0.022 |
| 31 | FBXO45 | 1.050 (1.003-1.099) | 0.035 |
| 32 | PSMB9 | 0.981 (0.964-0.998) | 0.032 |
| 33 | CCNB1 | 1.013 (1.001-1.024) | 0.032 |
| 34 | RAD51 | 1.075 (1.010-1.143) | 0.023 |
| 35 | PSMA7 | 1.003 (1.000-1.006) | 0.040 |
| 36 | DAXX | 0.967 (0.946-0.989) | 0.004 |
| 37 | YWHAZ | 1.002 (1.000-1.004) | 0.020 |
| 38 | PBX1 | 1.014 (1.002-1.026) | 0.022 |
| 39 | CD74 | 0.999 (0.999-1.000) | 0.001 |
| 40 | TNFRSF1B | 0.976 (0.954-0.998) | 0.030 |
| 41 | DCTPP1 | 1.017 (1.008-1.026) | 0.000 |
| 42 | CCND2 | 0.922 (0.865-0.984) | 0.014 |
| 43 | MAD2L1 | 1.047 (1.006-1.089) | 0.023 |
| 44 | SCD | 1.001 (1.000-1.002) | 0.022 |
| 45 | RAC2 | 0.979 (0.962-0.995) | 0.011 |
| 46 | NSMCE2 | 1.036 (1.003-1.071) | 0.034 |
| 47 | BATF | 0.977 (0.960-0.995) | 0.010 |
| 48 | ARMT1 | 1.003 (1.001-1.006) | 0.007 |
| 49 | TPT1 | 0.998 (0.996-0.999) | 0.001 |
| 50 | ITGAL | 0.952 (0.910-0.997) | 0.038 |
| 51 | SGCB | 1.031 (1.002-1.060) | 0.036 |

**Supplemental Table S4. Identified small molecular drugs by CMAP (Query 1.0)**

| **PubChem CID** | **Compound** | **Description** | **Raw_cs** | **Fdr_q_nlog10** |
| --- | --- | --- | --- | --- |
| 3032818 | Naproxol | Anti-inflammatory | -0.68 | 15.65 |
| 421610 | GANT-61 | GLI antagonist | -0.66 | 15.65 |
| 5311 | Vorinostat | HDAC inhibitor | -0.64 | 15.65 |
| 2756 | Cimetidine | Histamine receptor antagonist | -0.62 | 15.65 |
